# Cancer Association Study of Aminoacyl-tRNA Synthetase Signaling Network in Glioblastoma

**DOI:** 10.1371/journal.pone.0040960

**Published:** 2012-08-31

**Authors:** Yong-Wan Kim, ChangHyuk Kwon, Juinn-Lin Liu, Se Hoon Kim, Sunghoon Kim

**Affiliations:** 1 Catholic Research Institutes of Medical Science, College of Medicine, The Catholic University of Korea, Seoul, Korea; 2 Systems Biomedical Informatics National Core Research Center, Seoul National University, Seoul, Korea; 3 Brain Tumor Center, Department of Neuro-Oncology, The University of Texas M. D. Anderson Cancer Center, Houston, Texas, United States of America; 4 Department of Pathology, Yonsei University College of Medicine, Seoul, Korea; 5 Medicinal Bioconvergence Research Center, Seoul National University, Seoul, Korea; 6 WCU Department of Molecular Medicine and Biopharmaceutical Sciences, Seoul National University, Seoul, Korea; University of Georgia, United States of America

## Abstract

Aminoacyl-tRNA synthetases (ARSs) and ARS-interacting multifunctional proteins (AIMPs) exhibit remarkable functional versatility beyond their catalytic activities in protein synthesis. Their non-canonical functions have been pathologically linked to cancers. Here we described our integrative genome-wide analysis of ARSs to show cancer-associated activities in glioblastoma multiforme (GBM), the most aggressive malignant primary brain tumor. We first selected 23 ARS/AIMPs (together referred to as ARSN), 124 cancer-associated druggable target genes (DTGs) and 404 protein-protein interactors (PPIs) of ARSs using NCI’s cancer gene index. 254 GBM affymetrix microarray data in The Cancer Genome Atlas (TCGA) were used to identify the probe sets whose expression were most strongly correlated with survival (Kaplan-Meier plots versus survival times, log-rank t-test <0.05). The analysis identified 122 probe sets as survival signatures, including 5 of ARSN (VARS, QARS, CARS, NARS, FARS), and 115 of DTGs and PPIs (PARD3, RXRB, ATP5C1, HSP90AA1, CD44, THRA, TRAF2, KRT10, MED12, etc). Of note, 61 survival-related probes were differentially expressed in three different prognosis subgroups in GBM patients and showed correlation with established prognosis markers such as age and phenotypic molecular signatures. CARS and FARS also showed significantly higher association with different molecular networks in GBM patients. Taken together, our findings demonstrate evidence for an ARSN biology-dominant contribution in the biology of GBM.

## Introduction

Mammalian aminoacyl-tRNA synthetases (ARSs) and ARS-interacting multifunctional proteins (AIMPs) carry out the first step of protein synthesis by catalyzing the ligation of amino acids to their cognate tRNAs. However, they also contain other domains unrelated to catalytic activities to form diverse complexes with each other or with other cellular regulatory factors. This structural complexity seems to be linked to a functional versatility, and their expanded functions have been implicated in a variety of human diseases including cancers [1]. Several ARSs have been shown to be abnormally up- or down-regulated in hepatomas, colon cancer, Burkett’s lymphoma, prostate adenocarcinoma, breast cancer, sarcoma, colorectal adenocarcinoma and pituitary adenoma [2]–[5]. In addition, the functional regulation of cell growth, differentiation, RNA splicing, cytokine activities, and angiogenesis by ARSs in various disease states such as breast cancer, colon cancer, prostate cancer, and renal cell cancer has been studied [6]–[10]. Overexpression of MRS was also reported in malignant fibrous histiocytomas, sarcomas, malignant gliomas and glioblastomas [1]. These tumors have amplification of the chromosome 12q13 locus, where the gene for MRS overlaps with the gene for CHOP, which functions as an inhibitor of C/EBP12. This amplification probably results in the overexpression of MRS and CHOP, which may promote a favorable milieu for tumor progression [11]. However, despite that ARSs have been linked to human cancers, their biological significance is still not completely understood.

Here we described our integrative genome-wide analysis of ARSs to show cancer-associated regulatory activities with an emphasis on glioblastoma multiforme (GBM) [12] using NCI’s cancer gene index (CGI) [13] and The Cancer Genome Atlas (TCGA) database [14]. First, we selected 23 ARS/AIMPs (ARSN), 124 cancer-associated druggable target geneset (DTG) and 404 protein-protein interactors (PPIs) of ARSs. Then, we assigned each of the geneset to several prognostic molecular signatures of GBM (GEO accession #GSE4271) including proneural (PN), proliferative (Prolif) and mesenchymal (Mes) [15] and identified survival-related genes that are differentially expressed among samples in each subtype compared to other subtypes. We showed several candidates that are more likely to interact with aminoacyl-tRNA synthetase and their different involvement in each specific subtype. Thus, our study suggests potential contribution of ARSs with their PPI gene sets on the phenotype of GBM.

## Results

### ARSN Shows Potentially Association with Cancers through Interactions with DTGs

**Figure 1:**
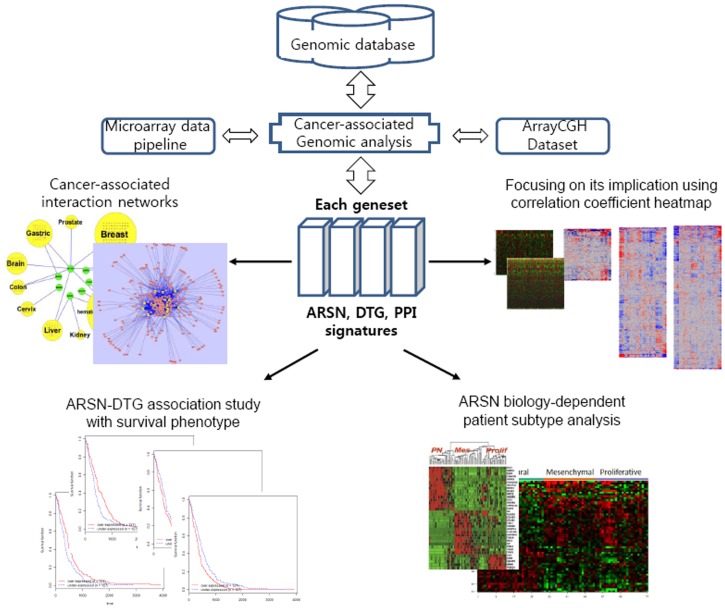
Outline of analysis procedures with each geneset showing the general steps required to identify genes that modulate a specific phenotype: selection of genes with the desired phenotype, and identification of phenotype-inducing ARSN and corresponding cancer-associated druggable target genes.

To examine the potential association of ARS family with cancer at a systemic level, we compared the expression profiles of the genes encoding the 20 human cytoplasmic ARSs and AIMP1–3 (AIMPs) with those of known DTGs obtained from the US National Cancer Institute’s cancer gene index (CGI). Detailed analysis procedures are outlined in Figure 1. We selected 124 DTGs that can interact with 23 ARS/AIMPs, and 404 genes as protein-protein interactors of ARSs. For the comparison, we also selected 1874 non-cancer-associated genes (nonCAGs) (each geneset is detailed in Table S1). In the first example from the CGI, the genesets were used to show cancer-associated regulatory activities with Cytoscape. We categorized the data into ten cancer groups (brain, colon, kidney, cervix, haematopoietic and lymphoid, liver, prostate, lung, breast, and gastric cancer).

**Figure 2:**
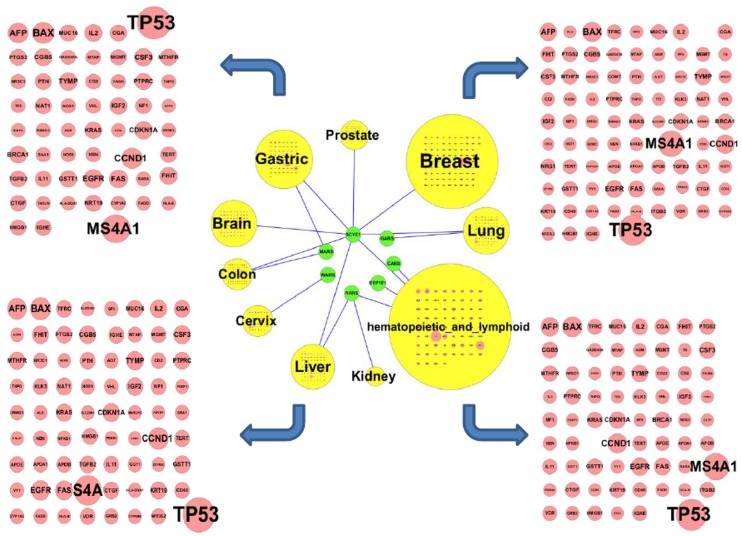
Cancer-associated interactions between 23 ARSs and AIMPs, and three genesets. 3501 genes were selected by manual curation, clinical examination and causal relationship to cancer. Using 11 public database showing the curated interactions of human proteins (HPRD, BioGRID, KEGG, Reactome, BIND, MINT, IntAct, InnateDB, DIP, STRING, and PharmDB), we further selected 124 DTGs and 404 genes as PPIs of ARSs. Using a cancer-associated interactions analysis, a cancer-association map was established to display how much ARSs and AIMPs could be differently interacted to ten different cancers. Each brown node indicates each gene of respective cancer and each node size indicates the degree of cancer-dependent co-association of a gene. Line indicates the co-association between ten cancers and seven ARSN. The cancer node size indicates the number of interactions with the brown node gene. Seven components of ARSN (green nodes) show relatively higher cancer-associated network.

Using the cancer-associated interactions between ARSs and AIMPs, and three genesets, as expected, a large number of the line based on the node of ARSN-DTGs indicates higher association between ARSN and DTGs than ARSN-PPIs (Figures S1, S2, S3, S4). A cancer-association map was also established to display how much ARSs and AIMPs could be differently interacted to ten different cancers (Figures S5, S6, S7, S8, S9, S10, S11, S12, S13, S14). Four large cancer sets connected to components of the cancer association interaction network were shown in Figure 2. The cancer node size indicates the number of interactions with the brown node gene. Among the components of ARSN, relatively higher cancer-associated network was shown by GARS, MARS, WARS, RARS, CARS, AIMP1 (SCYE1) and AIMP3 (EEF1E1) (green nodes). Also, AIMP1, MARS, and RARS have relatively higher association with cancers, indicating their potential importance in cancer biology and the needs of pathological mechanistic studies.

### Correlation Map Signatures Define ARSN Subclasses of Glioblastoma

**Figure 3:**
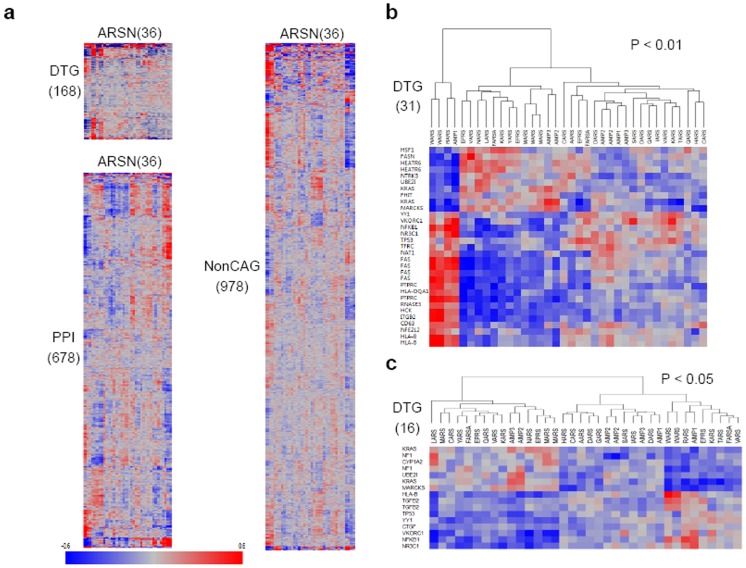
Correlation patterns of 23 ARSs and AIMPs to three different genesets. (**a**) We identified 846 resulting probe sets including 168 DTGs and 678 PPIs that can directly interact with ARSN using 254 GBM affymetrix U133plus2 microarray dataset in TCGA. For the comparison, we also selected 978 probe sets among 1874 nonCAGs. To understand ARSN-DTGs/PPIs/nonCAGs interactions and visualize the relationship between genesets, a correlation map was made on the basis of their correlation levels with each set. The probe sets are presented in matrix format, where rows represent individual genes of DTGs, PPIs, and nonCAGs, respectively, and columns represent each gene of ARSN. Each cell in the matrix represents the correlation level of a gene in an ARSN. Red color indicates that the gene tends to be up or down-regulated together; Blue color indicates the opposite tendency (The darker, the stronger the association between two genes). (**b**) Hierarchical clustering analysis showed that ARSN were shared by three groups with 31 DTGs (FDR <0.005). 31 DTGs were generated on a supervised hierarchical clustering analysis. (**c**) Hierarchical clustering of ARSN based on the 16 DTGs based on nonlinear association between two gene expression sets. 16 DTGs were correlated with three subgroups of ARSN.

For cancer-associated systematic analyses of ARSs and AIMPs, we applied the genesets to the 254 GBM affymetrix U133plus2 microarray dataset in the TCGA. In this dataset, we identified 846 resulting probe sets including 168 DTGs and 678 PPIs that can directly interact with ARSs and AIMP1–3. For comparison, we also selected 978 probe sets among 1874 nonCAGs (each probeset is detailed in Table S2). To understand ARSN interactions with each DTGs/PPIs/nonCAGs and visualize the relationship between the genesets, a correlation map was made. First, the DTGs probeset was clustered on the basis of Pearson correlation coefficients that related their expression patterns across the 254 GBM tissues to the expression patterns of ARSN over the same tissue set, as shown in Figure 3a. We then clustered PPIs and nonCAGs on the basis of these correlation coefficients. In this analysis, a red color indicates that two genes tend to be up or down-regulated together (positively); a blue color indicates the opposite tendency (negatively). The clustered map of the geneset-geneset correlation showed a relatively positive or negative correlation between two gene sets. For the three interaction sets in which the correlation coefficient rule for more than 0.4 performed significantly, we investigated the frequency of more than 0.4 to determine if any set had relatively high frequencies of significant correlation. The frequency histograms was shown in Figure S15. For the ARSN-DTG set, the correlation coefficient more than 0.4 had a relatively high frequency of 0.35% versus 0.51% of the ARSN-PPI. For the ARSN-nonCAG set, a relatively low frequency of 0.09% was shown. Also, the differences in the median values among the sets are statistically significant (P<0.001, one-way ANOVA).

To clearly understand molecular interactions of each geneset, we performed supervised hierarchical clustering analyses using the map, showing that three clusters lead to a low false discovery rate (FDR <0.005), thus appears to provide significant interactive specificities of each cluster (Figure 3b). ARSN were shared by three groups. The first ARSN subgroup appears to be closely related to DTGs. We identified that several ARSN such as WARS, RARS, and AIMP1 showed a highly significant association with 31 DTG genes (Table S3) (positive association with 21 DTGs and negative association with 10 DTGs), suggesting their potential contribution to the GBM biology. The second subgroup includes 11 ARSN (EPRS, VARS, NARS, LARS, FARS, KARS, YARS, MARS, AIMP3, and AIMP2) and showed reversed biological functions with 31 DTGs compared to the first group. However, the third group including 9 ARSN (AARS, DARS, SARS, GARS, IARS, TARS, QARS, HARS, and CARS) showed relatively low correlation coefficients. Current microarray data clustering methods are limited to linear association between individual gene expression values and phenotype. To understand nonlinear associations between gene expression and phenotype that may not be linear associations, we used a quantitative (numeric) trait analysis in which we could make relationships based on nonlinear association between two gene expression sets. This method is a useful tool for detecting gene classes correlated with a quantitative trait and to explore the patterns of gene-class association. In this analysis, 16 DTGs (Table S4) were correlated with three subgroups of ARSN (Figure 3c, P<0.05). The results of two association methods showed the same number of cluster with the similar trends of the associations. 9 DTGs (UBE2I, KRAS, MARCKS, NFKB1, NR3C1, TP53, VKORC1, YY1, and HLA–B) were overlapped for the linear and nonlinear associations. On the other hand, 4 genes (NF1, CYP1A2, TGFB2, and CTGF) were not detected as differentially interacted with ARSN by the linear association method. However, we couldn’t show if the nonlinear profiles reflect the general trends of the associations, so further studies are needed. A comparative analysis of the association pattern between the PPIs and the individual ARSN was shown in Figure S16 and S17. In this case, ARSN were shared by two groups, showing a highly positive and negative association with 119 PPI genes. But it showed a low false discovery rate (FDR <0.014, thus appears to provide weak interactive specificities of PPI as compared to DTGs. The nonlinear association analysis also showed two groups of ARSN having a highly significant association with 117 PPI genes. Taken together, these results indicated that ARSNs suggest possible mechanisms and processes involved in the DTGs regulation.

### ARSN Expression and Correlation with Survival in Patients with GBM

**Figure 4:**
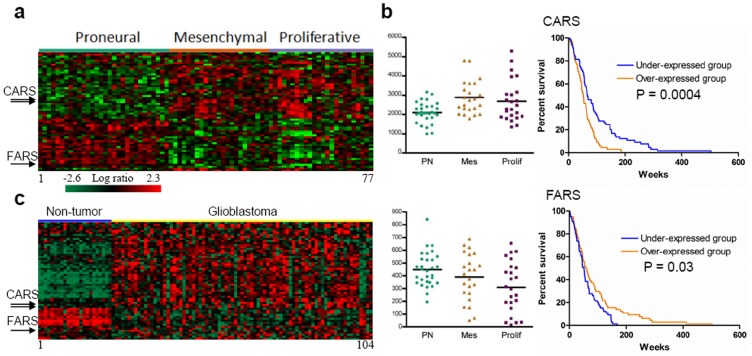
ARSN biology-dominant groups in patients with GBM. (**a**) We identified probe sets whose expression most strongly correlated with survival (Kaplan-Meier plots versus survival times, log-rank t-test <0.05). This analysis identified that 122 resulting probe sets of ARSN, DTGs, and PPIs that were correlated with survival in patients with GBM. Then, we performed a supervised clustering with the probesets and GBM subtypes such as proneural (PN), proliferative (Prolif) and mesenchymal (Mes). This analysis showed that 61 probeset as signature genes were differentially expressed in the three discrete subgroups. The 61 probe sets are presented in matrix format, where rows represent individual genes and columns represent each tissue. Each cell in the matrix represents the expression level of a gene in an individual tissue. Red and green cells reflect high and low expression levels, respectively. (**b**) Tumor subgroups are distinguished by CARS and FARS. Horizontal bars denote mean values. CARS is enriched in Mes and Prolif subgroups, while FARS in PN subgroup. Each Kaplan-Meier plot of overall survival in 130 GBM patients grouped on the basis of expression of CARS and FARS. The difference between two groups was significant when the P value was less than 0.05. (**c**) Hierarchical clustering of the GSE4290 dataset of 81 GBM samples from patients with GBM and 23 non-tumor tissues based on the 61 probe sets. Each gene with an expression status were shown in Supplementary Figure S21–S23. Nine probes were significantly overexpressed in the non-tumor samples, with 2 probes not showing in this analysis.

To identify gene expression patterns that classify GBM tumors into ARSN biology-dominant groups, we used 846 probe sets as described previously. We first identified probe sets whose expression most strongly correlated with survival (Kaplan-Meier plots versus survival times, log-rank t-test <0.05). This analysis identified that 122 resulting probe sets of ARSN (VARS, QARS, CARS, NARS, FARS), DTGs (PDE4A, NF1, NBN, CETP, SMAD3, HIST3H2A, TFRC, PTPRC, MTAP, etc), and PPIs (PARD3, RXRB, ATP5C1, HSP90AA1, CD44, THRA, TRAF2, KRT10, MED12, etc) that were correlated with survival in patients with GBM (Table S5). The effect of several genes expressions on survival was shown in Figures S18, S19, S20. Then, we performed a supervised clustering with the 122 probesets and GBM samples (GEO accession #GSE4271) showing well-known glioblastoma subtypes such as Proneural (PN; median survival of the PN subclass is 174.5 weeks), Proliferative (Prolif; 60.5 weeks) and Mesenchymal (Mes; 65.0 weeks) [15]. As shown in Figure 4a, this analysis showed that 61 probe sets among 122 probe sets were differentially expressed in the three discrete subgroups based on the statistical cut-off (P = 0.01), including CARS and FARS, and 59 probe sets (Table S6). Using 61 probeset as a signature, the PN subtype showed a dominant feature of expression pattern of the gene sets compared to Prolif and Mes subtypes, suggesting consistent prediction patterns by a 61 probe set expression signature. To determine whether CARS and FARS contribute to differences in biological characteristics of GBM tumors, we examined expression of two ARSs. As shown in Figure 4b, CARS was overexpressed in Prolif and Mes subtypes, while FARS was overexpressed in PN subtype. Kaplan-Meier plots and log-rank survival analyses showed that the median overall survival time of under-expressed CARS group was longer (66.9 weeks) than that of over-expressed group (51.4 weeks). Also, the median overall survival time of over-expressed FARS group was longer (59.2 weeks) than that of under-expressed group (50.0 weeks).

To search the expression of the 61 probe sets in normal brain tissues, we assessed the probe sets’ expression signatures with other independent public GBM gene expression datasets (e.g., GSE4290 dataset) [16]. In the GSE4290 dataset, the expression levels of the 61 probe sets were different between a set of non-tumors and GBM tumors (Figure 4c). CARS was overexpressed in the GBM tumors, while FARS was not significantly overexpressed in either tissue. Of interest, in the GSE4290 datasets for astrocytomas (II and III) and oligodendrogliomas (II and III), no correlations were identified with the 61 probe sets (Figures S21, S22, S23), suggesting that the 61 probe set signature was specific for GBM.

### CARS and FARS Correlations with different Molecular Networks in Patients with GBM

**Figure 5:**
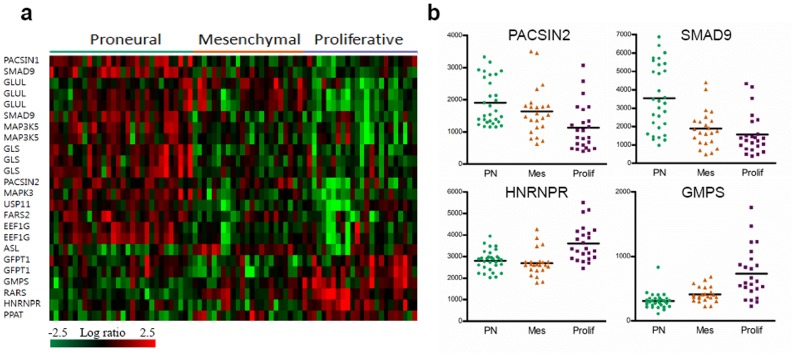
Molecular signatures of CARS and FARS interaction networks in patients with GBM. (**a**) We identified probe sets whose expression most strongly correlated with CARS and FARS in each subtype. This analysis identified that 88 resulting probe sets of the 48 genes. Then, we performed a supervised clustering with the probesets and GBM subtypes such as proneural (PN), proliferative (Prolif) and mesenchymal (Mes). This analysis showed that 24 probeset as signature genes were differentially expressed in the three discrete subgroups (P = 0.001). The 24 probe sets are presented in matrix format, where rows represent individual genes and columns represent each tissue. Each cell in the matrix represents the expression level of a gene in an individual tissue. Red and green cells reflect high and low expression levels, respectively. (**b**) Tumor subgroups are distinguished by interactors of CARS and FARS. Horizontal bars denote mean values.

To investigate if there is any difference in the interaction networks of CARS and FARS in the three subtypes, we selected 48 genes (Table S7) that can directly interact with CARS and FARS using our previously published data [1]. Then, we performed a supervised clustering with the 48 genes and GBM samples (GEO accession #GSE4271). As shown in Figure 5a, this analysis showed that 24 probes (16 genes) among 48 genes were differentially expressed in the three discrete subgroups (P = 0.001). In Figure 5b, several interactors, such as PACSIN2 and SMAD9, were overexpressed PN subtype, while HNRNPR and GMPS were overexpressed in Prolif subtype. Using 16 genes as a molecular interaction signature of CARS and FARS, the PN subtype showed a different feature of expression pattern of each interactor compared to Prolif and Mes subtypes, suggesting different interaction patterns of the two ARSs in each subtype. To detect the differences in the functional profiles, we placed differentially expressed genes in the context of present interactome knowledge, using the Ingenuity Pathways Analysis tools (*P* for all <0.05), showing that RNA metabolic process (FARSB, SMAD9, FARS2, MAPK3, HNRNPR, and RARS) was significantly related with PN and Prolif subtypes. Significantly up-regulated molecular functions for PN subtype was receptor signaling protein activity and protein phosphorylation pathway (SMAD9, MAPK3, and MAP3K5). This analysis showed that the molecular interaction differences of CARS and FARS in each subtype might be associated with differences in the clinical outcomes of GBM patients. Taken together, this study identified a highly interconnected network of aberrations, including 61 probe sets (59 PPIs and 2 ARSs) with the down-regulated CARS and the up-regulated FARS, which suggested that the difference of the interconnections might have important roles in long-term survival of patients in GBM. Thus, our results suggest potential contribution of ARSs with their interacting gene sets on the phenotype of GBM.

## Discussion

Recent evidence suggests that aminoacyl-tRNA synthetases (ARSs) exhibit remarkable functional versatility and their non-canonical functions have been pathologically linked to cancers. This study demonstrates that our integrative genome-wide analysis of ARSs shows cancer-associated activities in GBM and establishes 61 probe sets as survival signatures that are differentially expressed in the three different prognosis subgroups in patients with GBM. The interaction networks of CARS and FARS reveal the molecular interaction differences of CARS and FARS in each subtype, suggesting the potential differences in the clinical outcomes of GBM. This work suggests higher association with different molecular networks of an ARSs in the biology of GBM, and may yield key biological mechanisms behind the difference of the subtypes.

ARSs and AIMPs have been explored as therapeutic targets against cancer by network mapping criteria and known robust protein-protein interaction factors for most of these genesets, but these have been neglected due to the lack of correlations of genotype-phenotype in certain clinical situations [1]. The widespread acceptance that non-conventional functions of ARSs have been validated has spurred interest in investigating the reason that there are many diseases associated with ARSN [17], [18]. While numerous genetic alterations have been described in ARSN [18], [19], such markers have proved to be of non-essential in guiding disease complexities. Interestingly, recent expression profiling studies have revealed that regulatory molecular networks can be key factors to control tumorigenesis [1], [10], [20]. In the current study, we identified potential molecular interaction networks associated with tumor complexity as well as disease subclasses in GBM signaling pathways.

### ARSN Shows Cancer-associated Interactions

We compared the expression profiles of the genes encoding the 23 ARSN with DTGs obtained from the NCI’s cancer gene index (CGI) and established a cancer-association map showing how much ARSs and AIMPs could be differently interacted to ten different cancers. The strong association between ARSN and the ten cancers is consistent with our previous findings [1]. In our previous analysis, the 23 ARSN showed expression profiles that are similar to those of CAGs in ten different cancer types and that are clearly distinguishable from the pattern of nonCAGs (combined *P*<0.0001). ARSN showed a high degree of association in most of the tested cancers, except for pancreas, prostate, liver, and gastric cancers, which was consistent with our results. Reasons for association of ARSN with some cancer types (e.g., lymphomas, breast) but not others (e.g., pancreatic, prostate) should be further studied. Among the components of ARSN, in our two results, both GARS and AIMP1 showed relatively higher cancer-associated network, indicating their potential importance in cancer biology. While there has been progress in understanding the role of AIMP1 in cancer [21], [22], GARS functions in cancer biology have not been defined. Several previous studies have reported GRS-associated phenotypes that 11 distinct mutant alleles for GRS in the human population caused CMT (Charcot–Marie–Tooth) neuronal disease [19] and GRS upregulation in autoimmune patients [23] and defects in GARS are the cause of distal spinal muscular neuropathy type 5 [24]. Consistent with well-established correlations of GRS to several diseases, we already found that GRS proteins or fragments have activity to induce apoptosis of cancer cells specifically. In our previous study, the GRS proteins secreted from the macrophages was attached to cancer cells and involved in specific anticancer activities through caspase 3 activation and MAPK inactivation [25], [26]. While the current analysis utilizes a large scale analysis, these molecules are representative of a particular interest for new markers related to cancer treatments.

### Finding of ARSN Biology-associated Patient Subtype in Glioblastoma

For GBM-associated systematic analyses of ARSs and AIMPs, we performed ARSN-DTGs/PPIs/nonCAGs interaction analyses and visualized the relationship using several correlation maps, showing a significant positive or negative correlation between ARSN and other two gene sets. Pearson correlation coefficient is one of the most convenient measures to evaluate gene expression similarities. In our data set (254 GBM affymetrix U133plus2 microarray dataset), we used enough number of dataset because a smaller sample number tends to produce larger amplitude of correlation values between any two genes [27]. Also we did not integrate other dataset using public database because selection of the GeneChip normalization method strongly affected the performance of coexpression data. We used Pearson correlation coefficient itself to compare each dataset and showed the differences among the datasets statistically significant (P<0.001, one-way ANOVA). WARS, RARS, and AIMP1 in the first group showed highly associated correlation with 31 DTGs. While most ARSN members in the second group showed weak correlations, MARS in the third group was distinguished by markedly negative correlation with the DTGs. Two highly associated groups showed activation of ANSN and DTGs gene expression, indicative of protein biosynthesis and cell proliferation, respectively. Previous studies have suggested the overexpression of MRS in glioma and glioblastomas [11] but have not indicated the cause of the cancer. Of note, WRS- and MRS-guided groups are characterized by another signature analysis that has been associated with the patterns of gene-class association types. In this analysis, genes driving glioblastomas such as KRAS, NF1, MARCKS, TGFb2, TP53, and NFKB1 were differentially correlated with three classes of ARSN showing very similar groups to mentioned above [28]–[33]. These results indicated that ARSNs suggest possible mechanisms and processes involved in the cancer-associated regulation of GBM biology [34].

Several clinical information such as tumor stage and grade are powerful predictors of outcome in patients with cancers [35], [36]. Although these factors do not predict survival or response to therapy in patients with advanced disease [36], DNA microarray analyses have identified genes whose expression levels correlated with survival in cancers [37]. Several gene expression studies have identified prognostic gene sets using a statistical cut-off alone, but these genes have not been validated for more accurate diagnosis and prognosis. To identify clinically relevant gene expression profiles correlated with long-term survival and ARSN biology-dominant subgroups in GBM tumors, we first identified probe sets whose expression most strongly correlated with survival, showing 122 resulting probe sets in patients with GBM. Using well-known glioblastoma subtypes such as PN, Prolif and Mes [15], we found that a cluster of 61 signature genes were differentially expressed in the three discrete subgroups. The PN subtype showed a dominant feature of expression pattern of the 61 gene sets, while Prolif subtype appears similar to Mes subtype. The Prolif and Mes subtypes could appear to vary with the mere extent of expression of these 61 signatures compared to PN subtype and not diametrically oppositely regulated compared to 35 signature genes as reported previously [15]. However, the 61 probe sets were significantly correlated with survival among the 846 probe sets that can directly interact with ARSN, and differentially expressed in the three discrete subgroups based on the statistical cut-off (P = 0.01). This study thus suggests that the molecular interaction differences of ARSN in each subtype might be associated with differences in the clinical outcomes of GBM patients. Also, a highly interconnected network of the 61 probe sets might correlate with the established better survival markers of the PN such as a younger age and grade III-like histology [15]. Of note, the expression of CARS and FARS appears to be unique to tumors of the PN subtype. We then used the interaction networks of CARS and FARS to explore the molecular interaction differences of CARS and FARS in each subtype, suggesting the potential differences in the clinical outcomes of GBM patients. Our protein interaction network-based approach is to explore inter-connected proteins responsible for specific cellular functions [38]. Using the PPI networks, the identified disease-related genes could be functionally related and reveal key biological mechanisms behind the difference of the subtypes [39], [40].

The significant association between CARS and FARS, and the PN signature supports our classification, linking the existence of an ARSN biology-dominant subgroup, not described in other studies. Thus, the 61 gene signatures might correlate with the established survival markers of the PN [41], but in this study we could not show a clear hypothesis to explain the relationship two poor prognosis subtypes with the signatures. Thus, further studies and reproducible results are necessary to evaluate an ARSN biology-dominant subgroup in the biology of GBM. Also, understanding whether any of these targets are driver genes of aberrant tumor growth and survival potency in GBM is a next major challenge of our research. Taken together, our results suggest potential contribution of ARSN with their interacting DTG and PPI gene sets on the phenotype of GBM. Our findings provide the rational basis for the development of new drug leads and therapeutic concepts in the ARSN studies [42], [43].

## Materials and Methods

### Geneset Selection

We selected 4 different genesets, as published previously [1], to examine the potential association of the ARSs and AIMP1–3 (together referred to as ARSN) with GBM at a systemic level. Briefly, we used the NCI cancer gene index, a collection of records on 6,955 human genes that were selected from Medline searches using cancer-related disease or drug/compound terms in the NCI Thesaurus (https://cabig.nci.nih.gov/inventory/data-resources/cancer-gene-index). From these genes, we selected 3501 cancer-associated genes that have been validated by manual curation, and causal relationship to cancer. Using 11 public database showing the curated interactions of human proteins (HPRD, BioGRID, KEGG, Reactome, BIND, MINT, IntAct, InnateDB, DIP, STRING, and PharmDB), we further selected 528 protein-protein interactors with 23 ARS/AIMPs. Among those genes, we selected 124 DTGs that consistently up- or down-regulated in 43 GEO sets (www.ncbi.nlm.nih.gov/geo) of 14 different cancer types, and 404 genes as protein-protein interactors (PPIs) of ARSs. The curated interactions were defined as the ones identified by various assays including yeast two hybrid and immunoprecipitation. For the comparison, we also selected 1874 non-cancer-associated genes (nonCAGs) that are not included in the CGI and do not show significant cancer-associated expression profiles in 22 public cancer datasets (P values >0.5) as reported previously [1]. These genes may interact with ARSN but not implicated in cancer.

### Glioblastoma Dataset in The Cancer Genome Atlas

To analyze the expression data, we directly accessed the input data through TCGA Data Portal (254 GBM affymetrix U133plus2 expression array). The CEL files were re-processed using the R statistical computing platform and packages from Bioconductor bioinformatics software project (www.r-project.org), and a RMA (robust multiarray average) intensity on a log-squared scale was generated for each probe set. Two independent filters were applied to probesets to remove low level signal intensity or not expression in brain tissue: 1) Probesets with less than 10% “present” and “marginal” calls were removed. 2) Probesets that contains more than 10% of accumulated zeros across samples were removed. These steps reduced the probesets from 54,000 to 34,178 in the set. Genes that have significantly changed can then be analyzed further. A t-test assesses whether the means of two groups are statistically different from each other. Then we performed supervised hierarchical clustering based on the most variably expressed genes using the Euclidean distance as the similarity metric and the complete linkage method as the between-cluster distance metric. We analyzed the functional networks using tools from Cytoscape that is an open-source software for visualizing molecular interaction networks [44]. To validate the data generated by TCGA, we directly accessed another independent public GBM gene expression datasets (GEO accession #GSE4271) [15]. In total, 100 tumors having survival clinical data were profiled for class discovery and survival analysis. Survival was defined as the time interval from surgery until the date of death.

### Correlation-coefficient Map Construction

To calculate the degree of association between ARSN geneset and other three genesets on the basis of their gene expression, we calculated correlation coefficient as follows [45]–[47]; we normalized each expression level of one probeset by subtracting its row-wise mean and dividing by its row-wise standard deviation; normalized each expression level of another probeset by subtracting its row-wise mean and dividing by its row-wise standard deviation. Then we took the inner product of the one normalized probeset and the transpose of the normalized another probeset; and divided each element in the resulting matrix by the number of microarray minus one. The resulting correlation coefficient matrix contains Pearson correlation coefficients relating an association pattern in the ARSN expression and other three probesets expression. Each probeset was then clustered on the basis of Pearson correlation coefficients that related their expression patterns across the 254 GBM tissues to the expression patterns of ARSs over the same tissue set. We then clustered PPIs and nonCAGs over ARSN on the basis of these correlation coefficients. Hierarchical clustering (GENE CLUSTER v3.0) and display programs (TREE VIEW) were used for analysis (http://rana.stanford.edu/software). We performed unsupervised hierarchical clustering based on the most variably expressed genes using the Euclidean distance as the similarity metric and the average linkage method as the between-cluster distance metric. Supervised clustering of experimental samples was performed by reducing the number of genes by statistical analysis.

### Survival-associated Probeset Analysis

To identify probe sets whose expression most strongly correlated with survival, samples were assigned into two groups based on the expression of each probeset such as a low expression group and a high expression group. We then performed Kaplan-Meier survival analysis (Kaplan-Meier plots versus survival times, log-rank t-test <0.05) and estimated the survival distributions and the log-rank test to assess the statistical significance of the differences between the stratified survival groups using GraphPad Prism (version 5, GraphPad Software Inc., San Diego, CA) [48]. Then, we assigned each of the samples to three well-known glioblastoma subtypes [15] by hierarchical clustering using the resulting survival-associated probe sets that were selected as mentioned above. A t-test (p<0.01) was used to identify marker genes whose expression differed between samples in each subtype class compared to other subtypes. For each subtype, differentially expressed class signatures were compared to discover a degree of difference. To verify class signatures in independent samples, expression profiles of GBM samples were used [49] and predicted the subtype of the samples in this validation dataset.

### Pathway Analyses

Genes that showed differences in their expression levels were selected for the different analyses (functional cluster analysis and biological pathway analysis). To classify the pathway profiles, functional analyses and KEGG (Kyoto Encyclopedia for Genes and Genomes) pathway analyses (http://www.genome.jp/kegg/pathway.html) were carried out as previously described [50], [51]. To perform a KEGG analysis, differentially expressed genes of each subtype were used for the calculation of their attribution to pre-defined KEGG signaling pathways and analyzed by pair-wise comparisons. The different number of genes were seen in a given pathway. The Ingenuity Pathway Analysis software (IPA, Ingenuity Systems, Mountain View, CA) was utilized to identify networks of interacting genes and other functional groups. Semantically consistent pathway relationships were modeled based on a continual, formal extraction from the public domain literature (www.ingenuity.com/products/pathways_ knowledge.html).

## Supporting Information

Figure S1 **Cancer-associated interactions between ARSs and AIMPs, and PPI.** We selected 124 DTGs that can significantly interact with 23 ARS/AIMPs, and 404 genes as PPIs of ARSs. For the comparison, we also selected 1874 non-cancer-associated genes (nonCAGs). Each brown node indicates each gene of the geneset. Line indicates the co-association with ARSN.
(TIF)

Figure S2 **Cancer-associated interactions between ARSs and AIMPs, and DTGs.**
(TIF)

Figure S3 **Cancer-associated interactions between ARSs and AIMPs, and nonCAGs.**
(TIF)

Figure S4 **Numbers of the cancer-associated interactions between ARSs and each geneset.** A large number of the line based on the node of ARSN-DTGs indicates higher association between ARSN and DTGs than ARSN-PPIs.
(TIF)

Figure S5 **A cancer-association map of DTGs in brain cancer.** Using a cancer-associated interactions analysis, a cancer-association map was established to display how much each DTG gene could be differently interacted to ten different cancers. Each brown node indicates each gene of the DTGs and node size indicates the degree of cancer-dependent co-association of the gene.
(TIF)

Figure S6 **A cancer-association map of DTGs in breast cancer.**
(TIF)

Figure S7 **A cancer-association map of DTGs in cervical cancer.**
(TIF)

Figure S8 **A cancer-association map of DTGs in colon cancer.**
(TIF)

Figure S9 **A cancer-association map of DTGs in gastric cancer.**
(TIF)

Figure S10 **A cancer-association map of DTGs in hematopoietic and lymphatic cancer.**
(TIF)

Figure S11 **A cancer-association map of DTGs in renal cancer.**
(TIF)

Figure S12 **A cancer-association map of DTGs in liver cancer.**
(TIF)

Figure S13 **A cancer-association map of DTGs in lung cancer.**
(TIF)

Figure S14 **A cancer-association map of DTGs in prostate cancer.**
(TIF)

Figure S15 **Histogram showing the frequency of the correlation coefficient.** The histogram was computed from the three interaction set. Bars represent number of correlation coefficients within the range indicated on the x-axis (P<0.001, one-way ANOVA).
(TIF)

Figure S16 **Correlation patterns of 23 ARSs and AIMPs to PPI.** Hierarchical clustering analysis showed that ARSN were shared by two groups with 119 PPIs (FDR <0.014). 119 PPIs were generated on a supervised hierarchical clustering analysis.
(TIF)

Figure S17 **Correlation patterns of 23 ARSs and AIMPs to PPI.** Hierarchical clustering of ARSN based on the 117 DTGs based on nonlinear association between two gene expression sets. 117 PPIs were correlated with two subgroups of ARSN.
(TIF)

Figure S18 **Effect of ARSN gene expression on survival in 254 GBM patients.** Kaplan-Meier plot of overall survival in 254 GBM patients grouped on the basis of expression of each probeset. The difference between two groups was significant when the P value was less than 0.05.
(TIF)

Figure S19 **Effect of DTG gene expression on survival in 254 GBM patients.**
(TIF)

Figure S20 **Effect of PPI gene expression on survival in 254 GBM patients.**
(TIF)

Figure S21 **Hierarchical clustering of the GSE4290 dataset.** Hierarchical clustering of the GSE4290 dataset of 81 GBM samples from patients with GBM and 23 non-tumor tissues based on the 61 probe sets. Nine probes were significantly overexpressed in the non-tumor samples, with 2 probes not showing in this analysis. The data are presented in matrix format in which rows represent individual genes and columns represent each tissue. Each cell in the matrix represents the expression level of a gene feature in an individual tissue. Red and green in cells reflect high and low expression levels, respectively.
(TIF)

Figure S22 **Hierarchical clustering of the GSE4290 dataset of astrocytomas (II and III).**
(TIF)

Figure S23 **Hierarchical clustering of the GSE4290 data set of oligodendrogliomas (II and III).**
(TIF)

Table S1 **Four gene sets including ARSN, DTG, PPI, and nonCAG.**
(XLS)

Table S2 **Probe sets of each ARSN, DTG, PPI, and nonCAG.**
(XLS)

Table S3 **31 DTG genes associated with ARSN.**
(XLS)

Table S4 **16 DTGs correlated with three subgroups of ARSN.**
(XLS)

Table S5 **122 probe sets correlated with survival in patients with GBM.**
(XLS)

Table S6 **61 probe sets among 122 probe sets.**
(XLS)

Table S7 **48 genes that can directly interact with CARS and FARS.**
(XLS)
