# Supplementary figures and images for: Cancer Association Study of Aminoacyl-tRNA Synthetase Signaling Network in Glioblastoma
Source: PLoS One. 2012 Aug 31;7(8):e40960. doi: 10.1371/journal.pone.0040960 (PMC3432027; doi:10.1371/journal.pone.0040960)

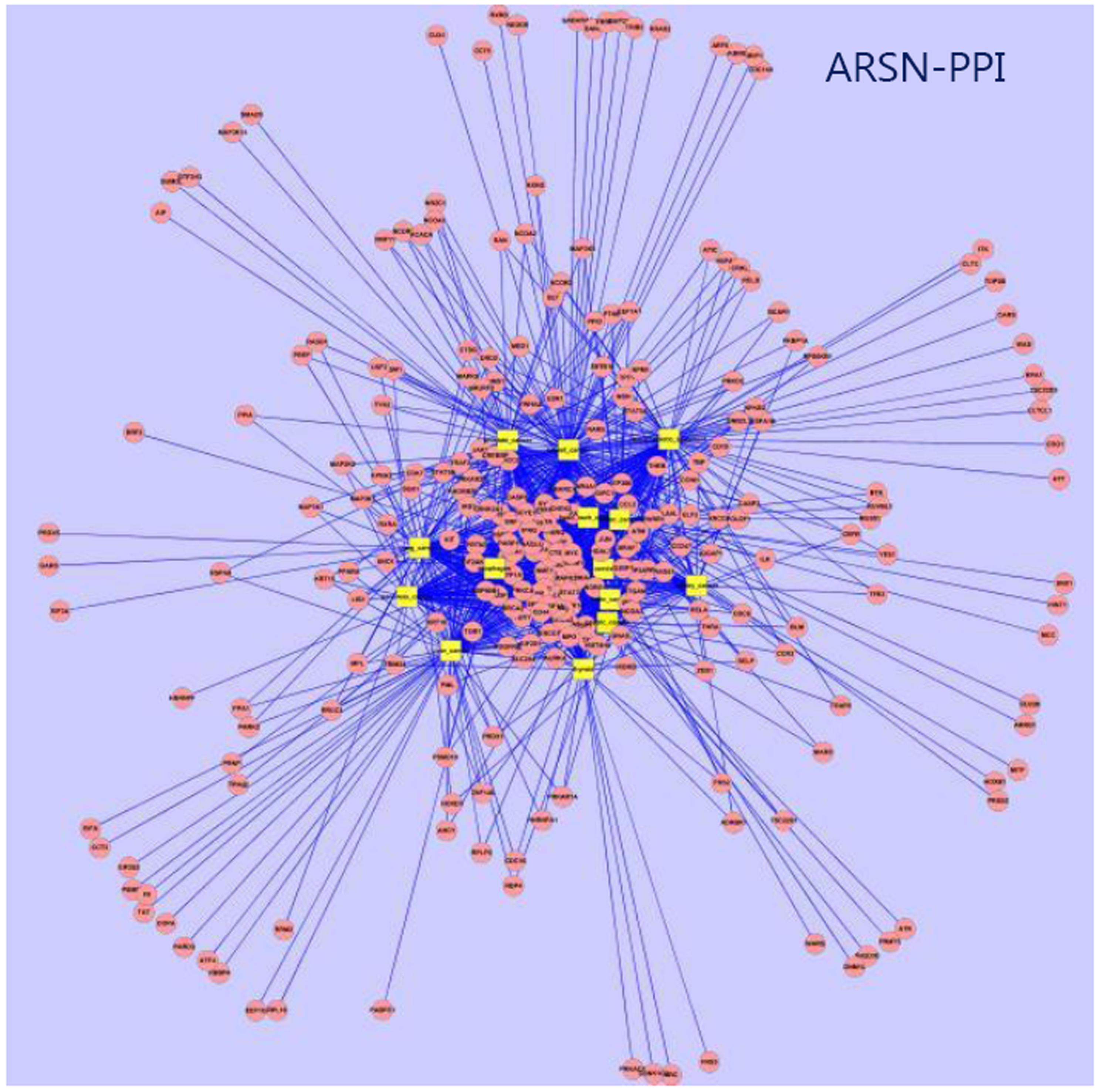

Supplement: Figure S1 — Cancer-associated interactions between ARSs and AIMPs, and PPI. We selected 124 DTGs that can significantly interact with 23 ARS/AIMPs, and 404 genes as PPIs of ARSs. For the comparison, we also selected 1874 non-cancer-associated genes (nonCAGs). Each brown node indicates each gene of the geneset. Line indicates the co-association with ARSN. (TIF) [file pone.0040960.s001.tif]

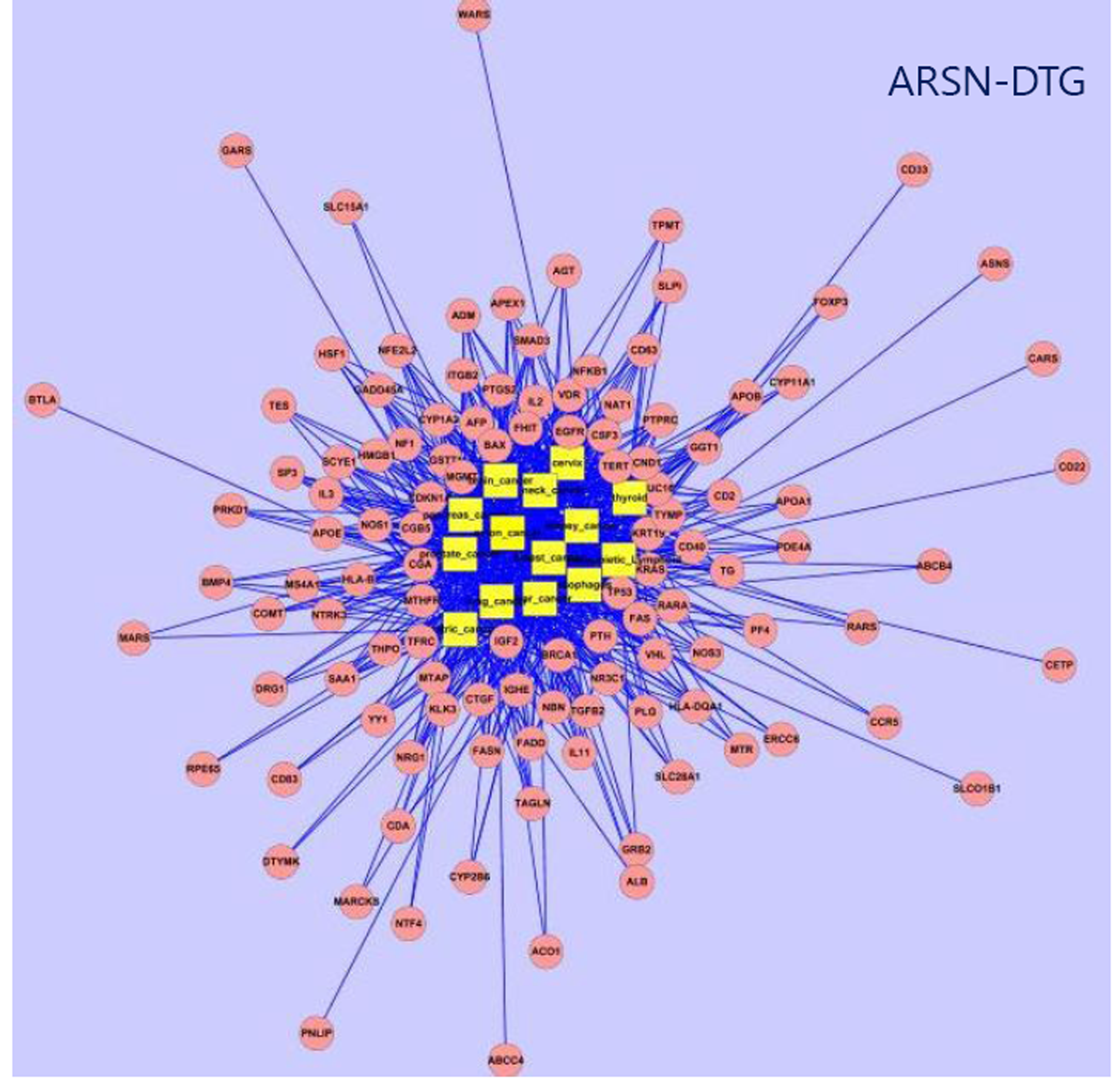

Supplement: Figure S2 — Cancer-associated interactions between ARSs and AIMPs, and DTGs. (TIF) [file pone.0040960.s002.tif]

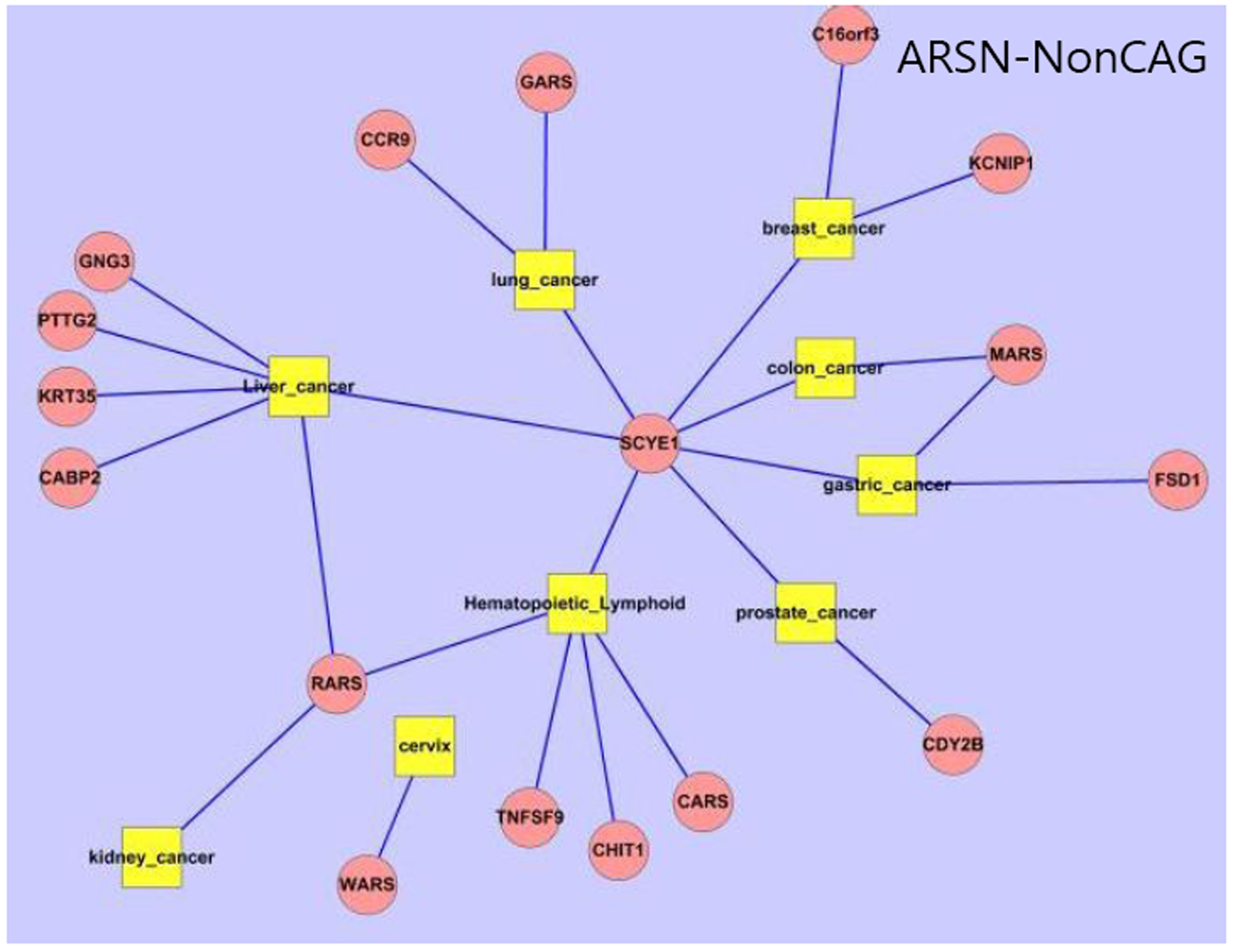

Supplement: Figure S3 — Cancer-associated interactions between ARSs and AIMPs, and nonCAGs. (TIF) [file pone.0040960.s003.tif]

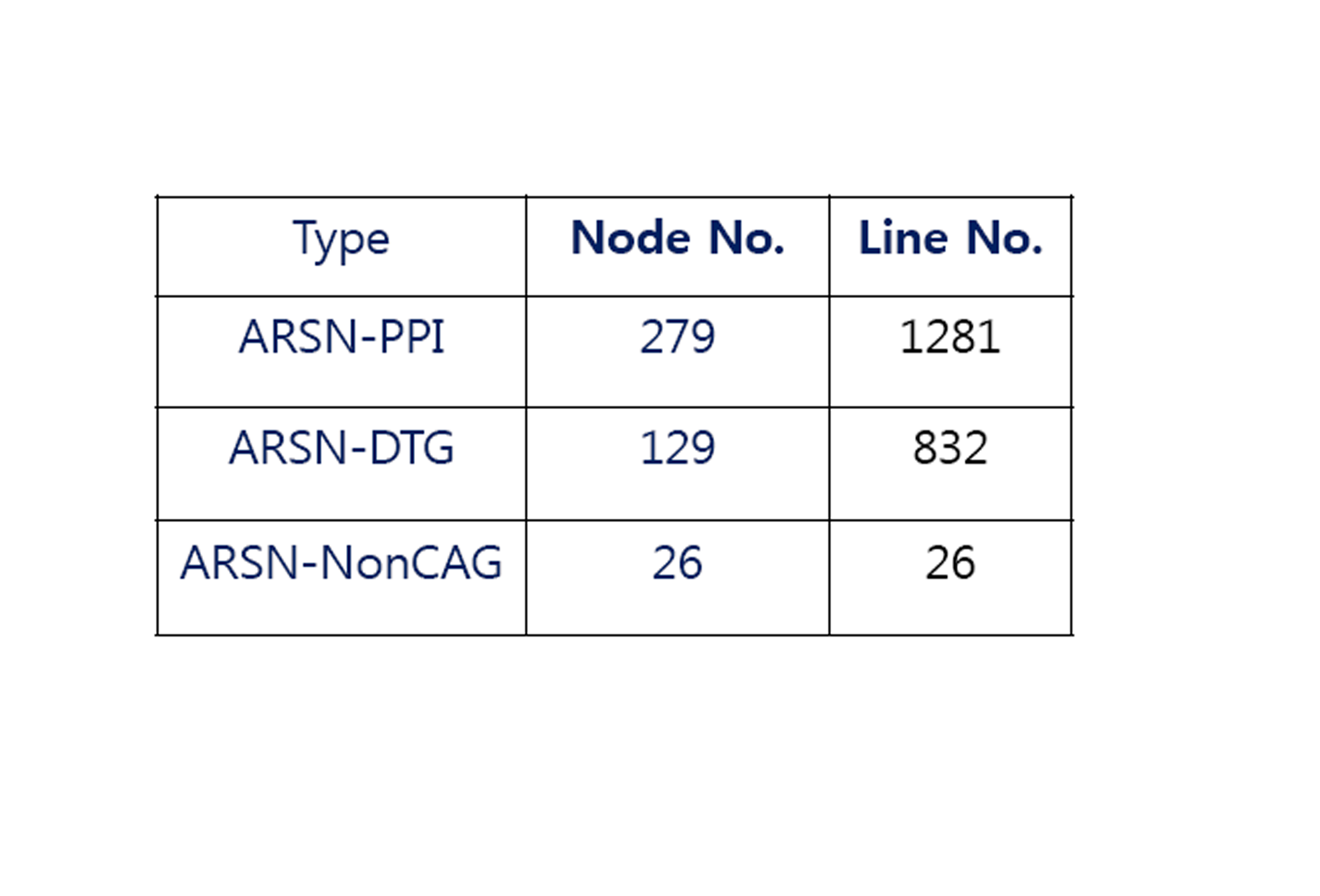

Supplement: Figure S4 — Numbers of the cancer-associated interactions between ARSs and each geneset. A large number of the line based on the node of ARSN-DTGs indicates higher association between ARSN and DTGs than ARSN-PPIs. (TIF) [file pone.0040960.s004.tif]

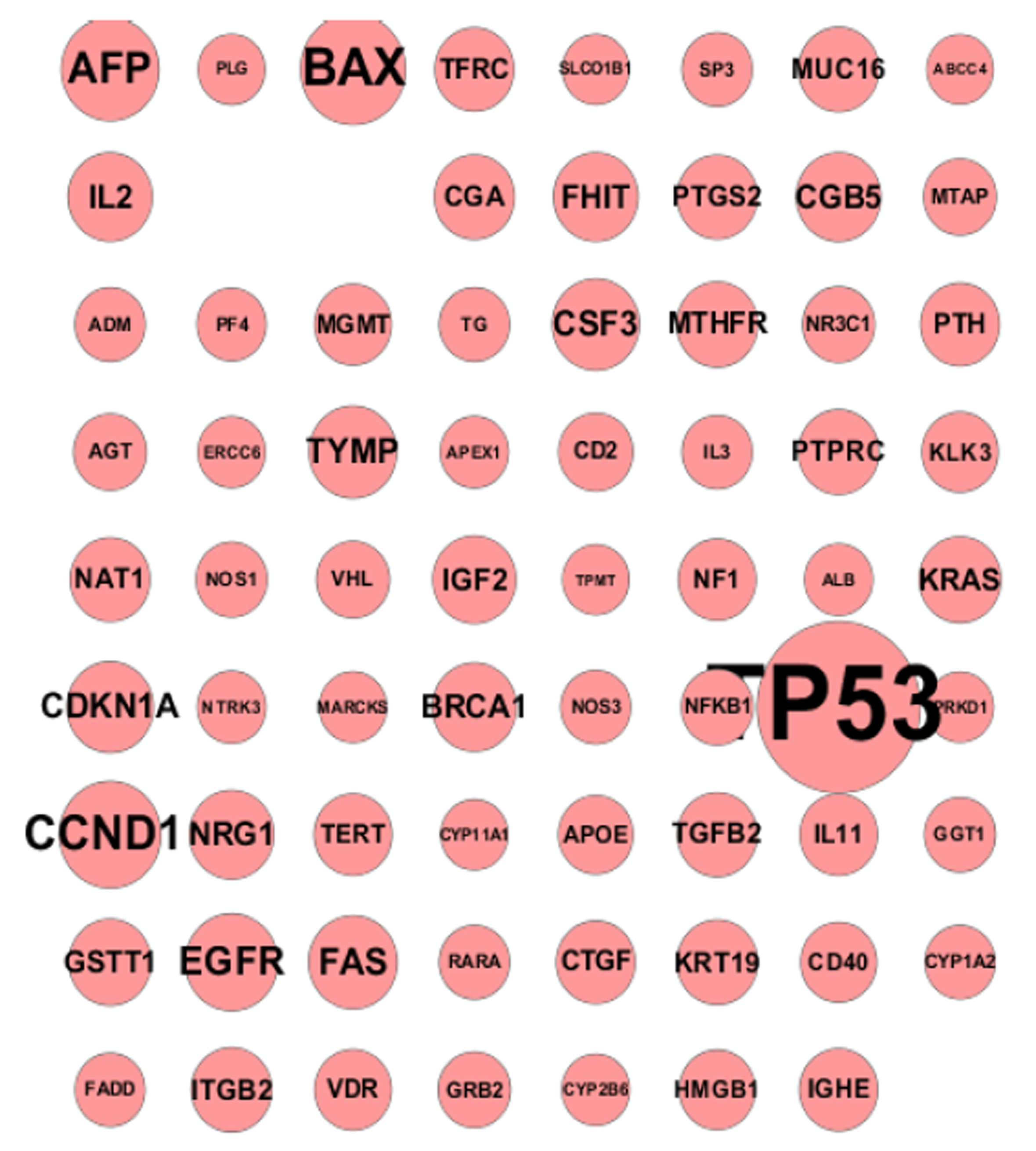

Supplement: Figure S5 — A cancer-association map of DTGs in brain cancer. Using a cancer-associated interactions analysis, a cancer-association map was established to display how much each DTG gene could be differently interacted to ten different cancers. Each brown node indicates each gene of the DTGs and node size indicates the degree of cancer-dependent co-association of the gene. (TIF) [file pone.0040960.s005.tif]

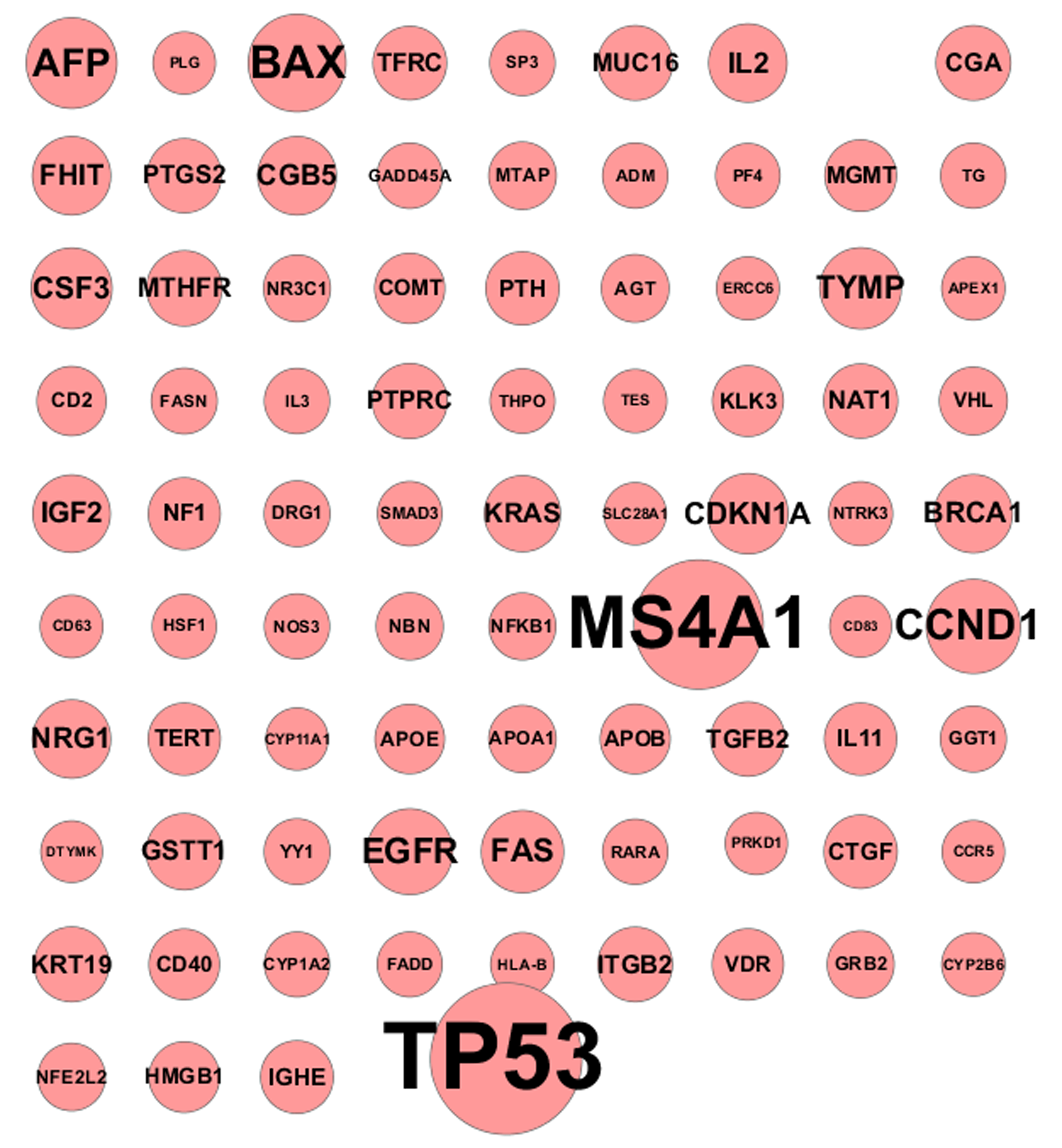

Supplement: Figure S6 — A cancer-association map of DTGs in breast cancer. (TIF) [file pone.0040960.s006.tif]

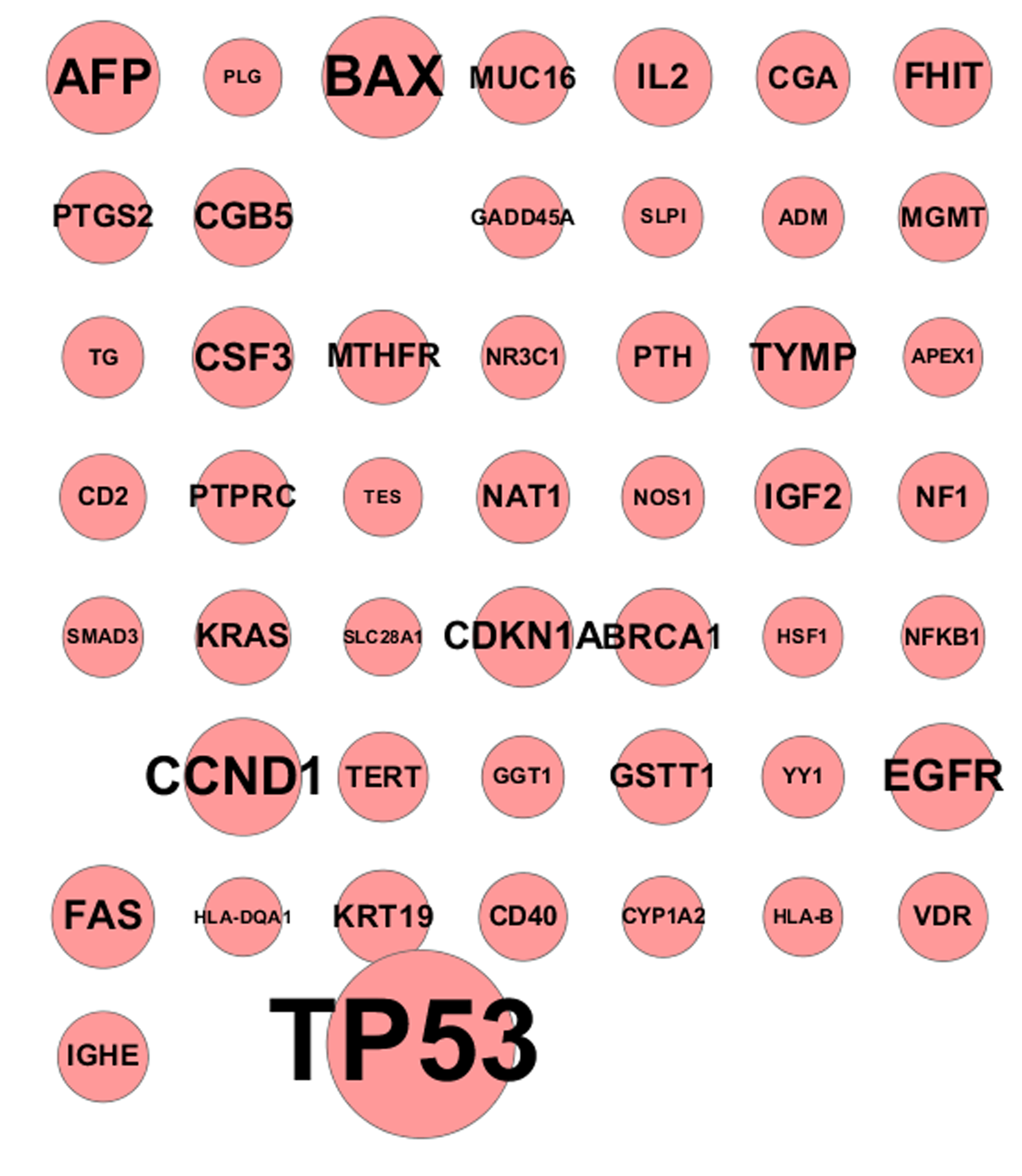

Supplement: Figure S7 — A cancer-association map of DTGs in cervical cancer. (TIF) [file pone.0040960.s007.tif]

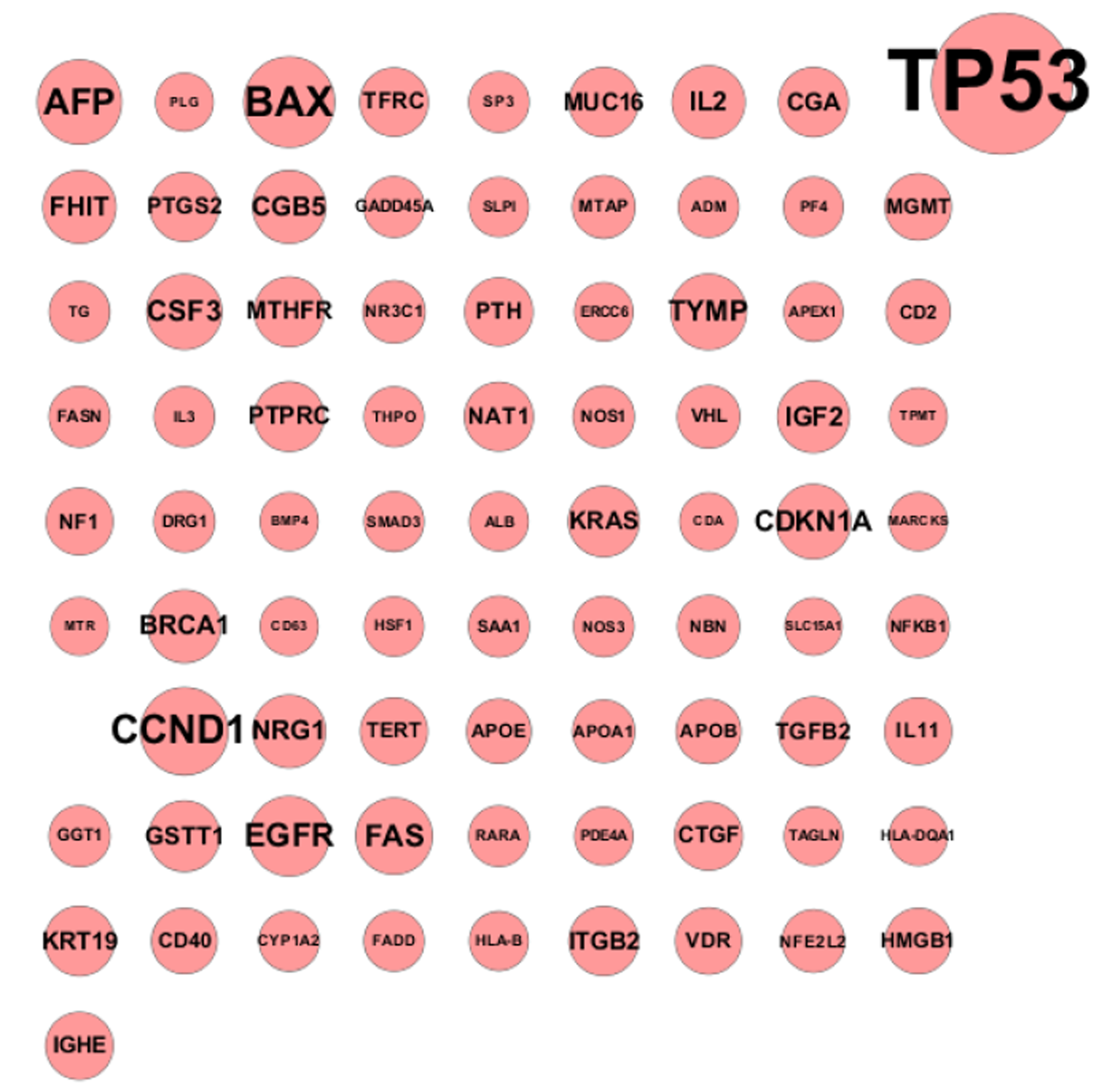

Supplement: Figure S8 — A cancer-association map of DTGs in colon cancer. (TIF) [file pone.0040960.s008.tif]

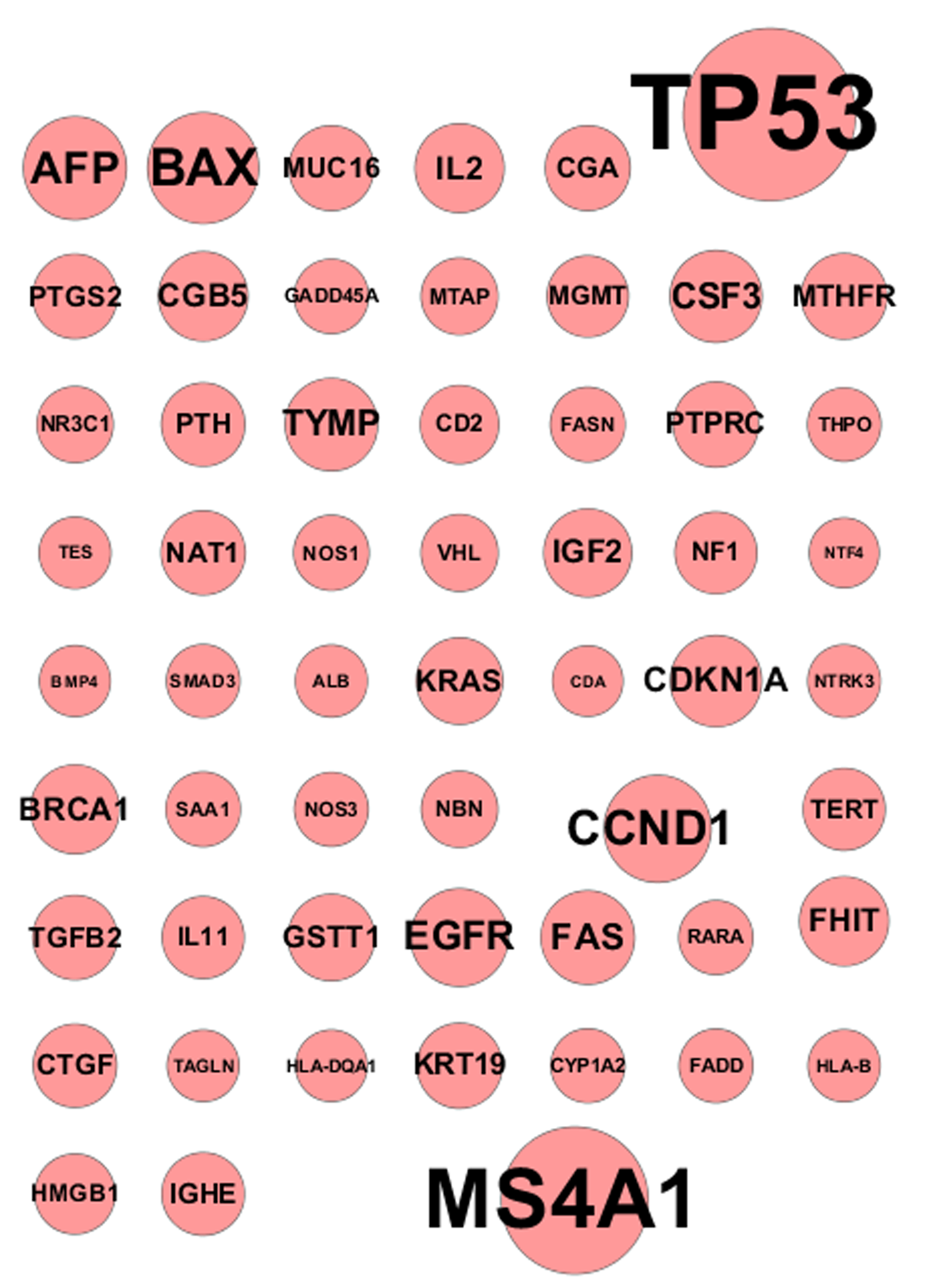

Supplement: Figure S9 — A cancer-association map of DTGs in gastric cancer. (TIF) [file pone.0040960.s009.tif]

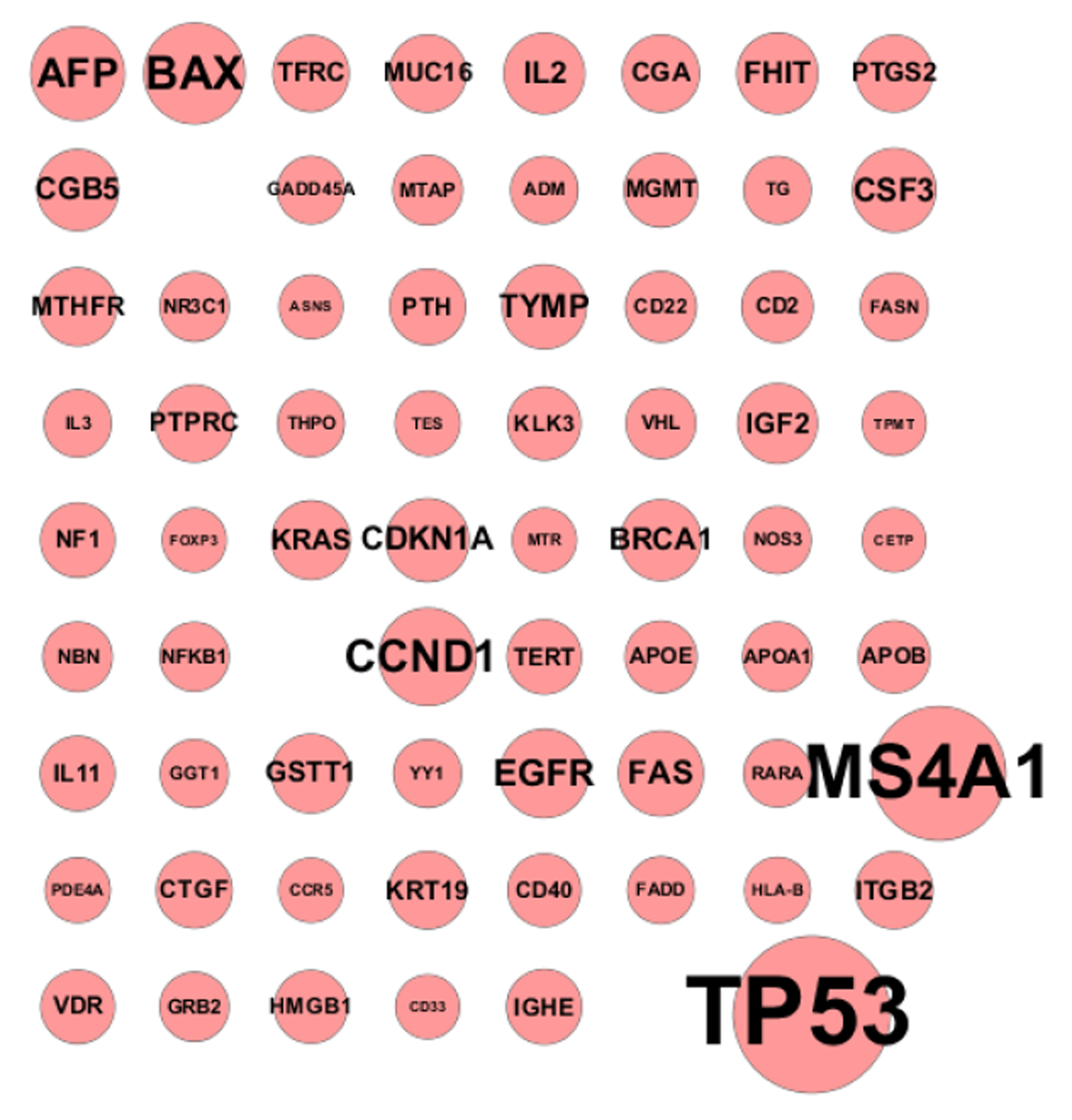

Supplement: Figure S10 — A cancer-association map of DTGs in hematopoietic and lymphatic cancer. (TIF) [file pone.0040960.s010.tif]

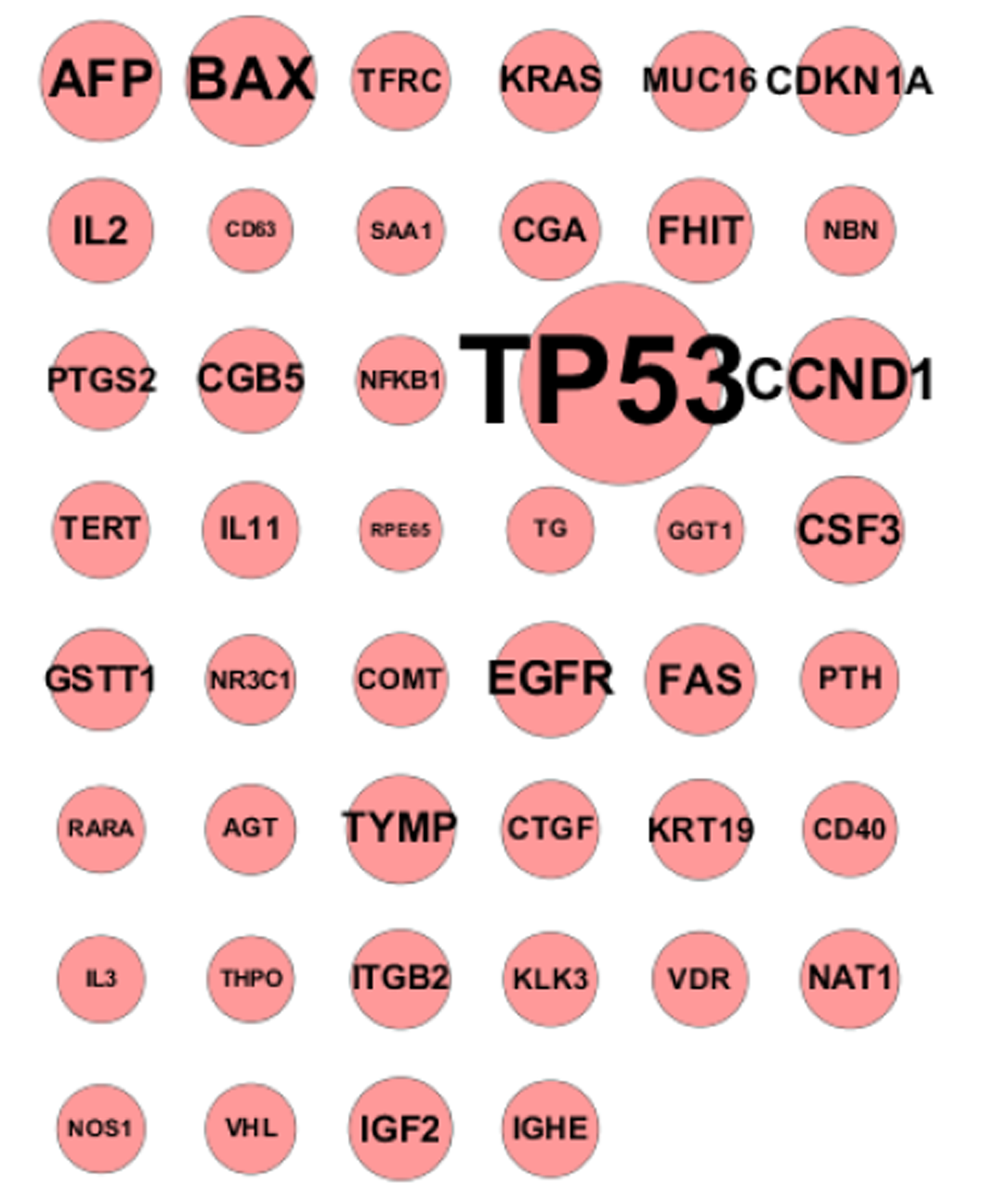

Supplement: Figure S11 — A cancer-association map of DTGs in renal cancer. (TIF) [file pone.0040960.s011.tif]

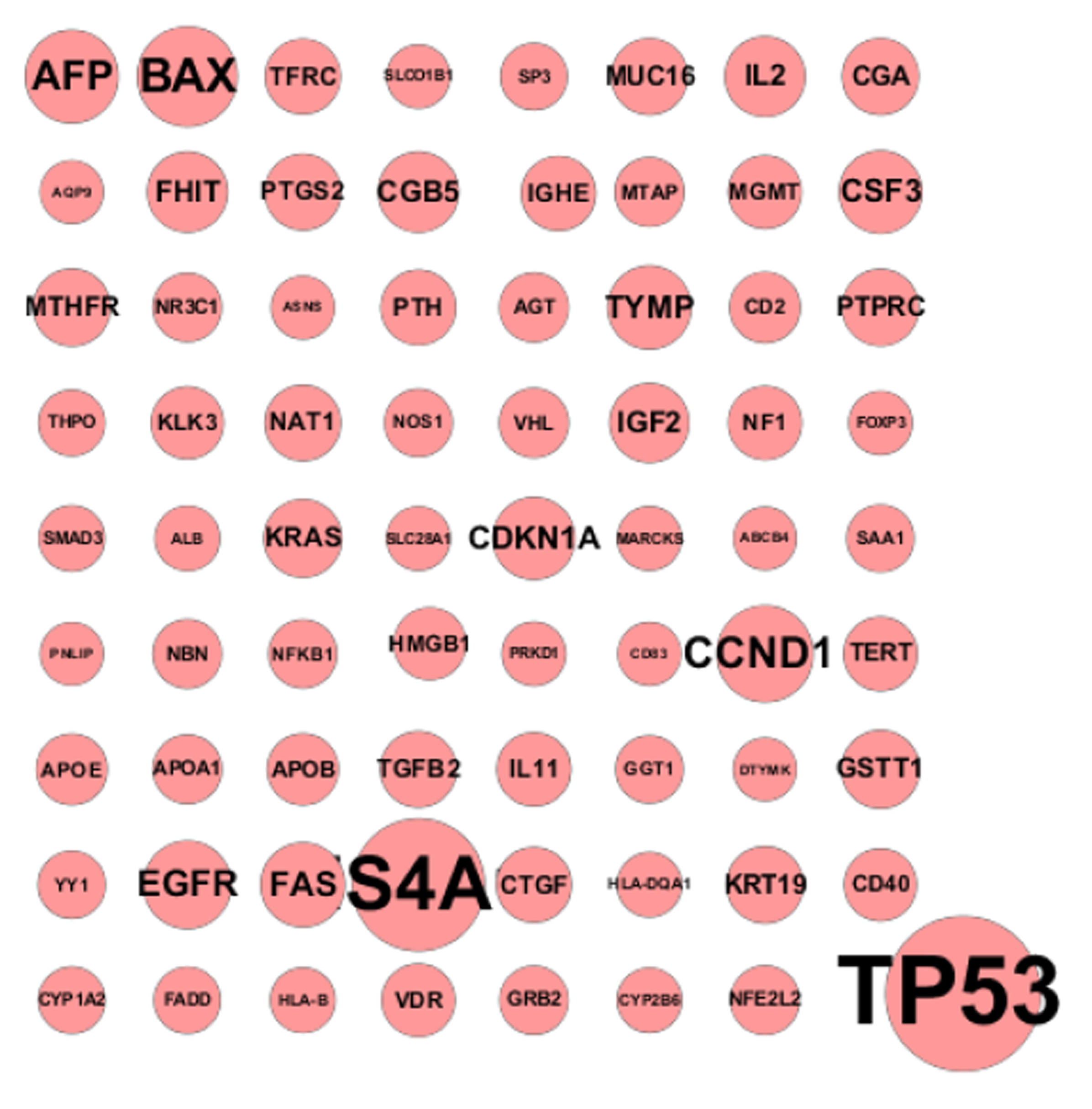

Supplement: Figure S12 — A cancer-association map of DTGs in liver cancer. (TIF) [file pone.0040960.s012.tif]

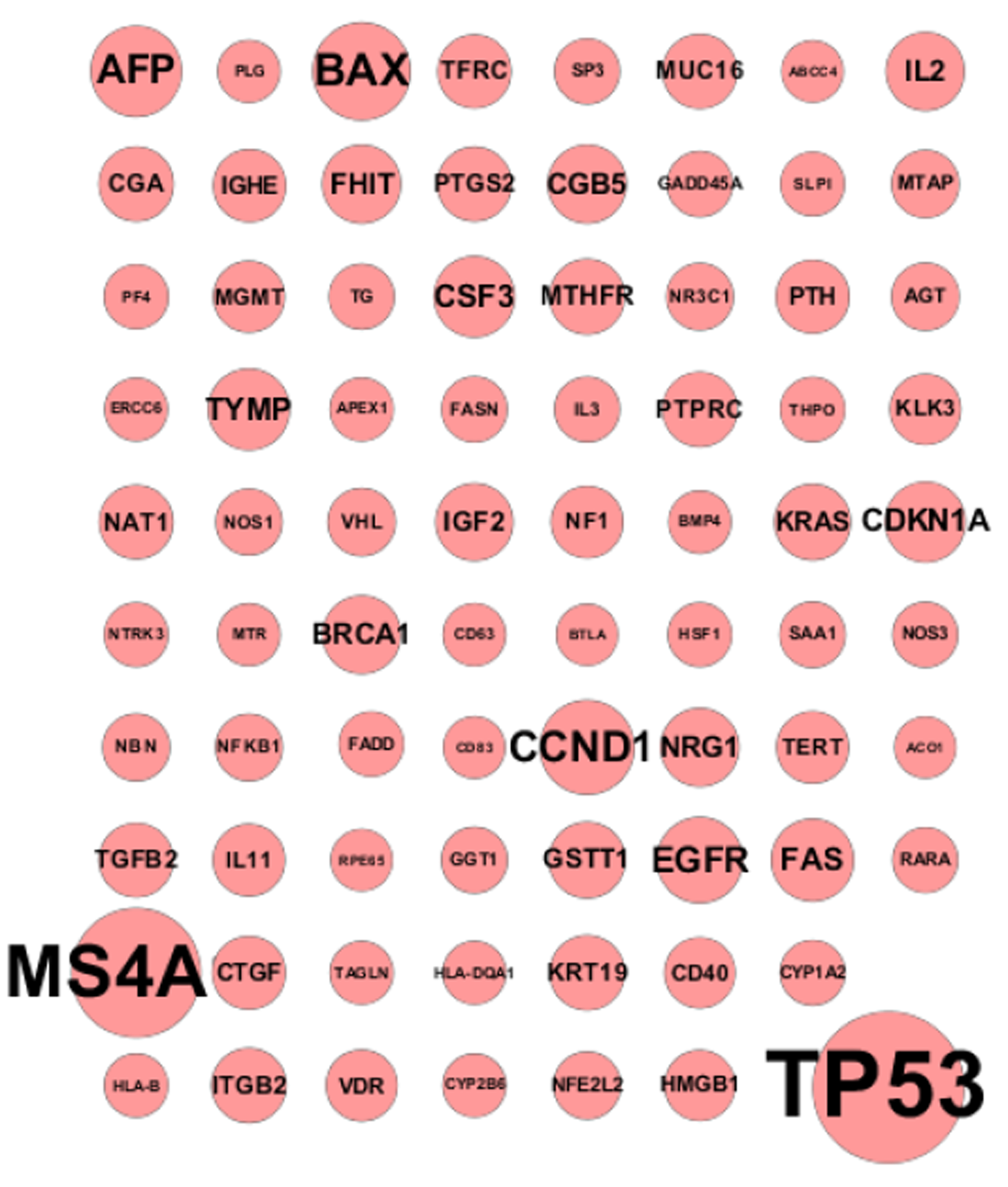

Supplement: Figure S13 — A cancer-association map of DTGs in lung cancer. (TIF) [file pone.0040960.s013.tif]

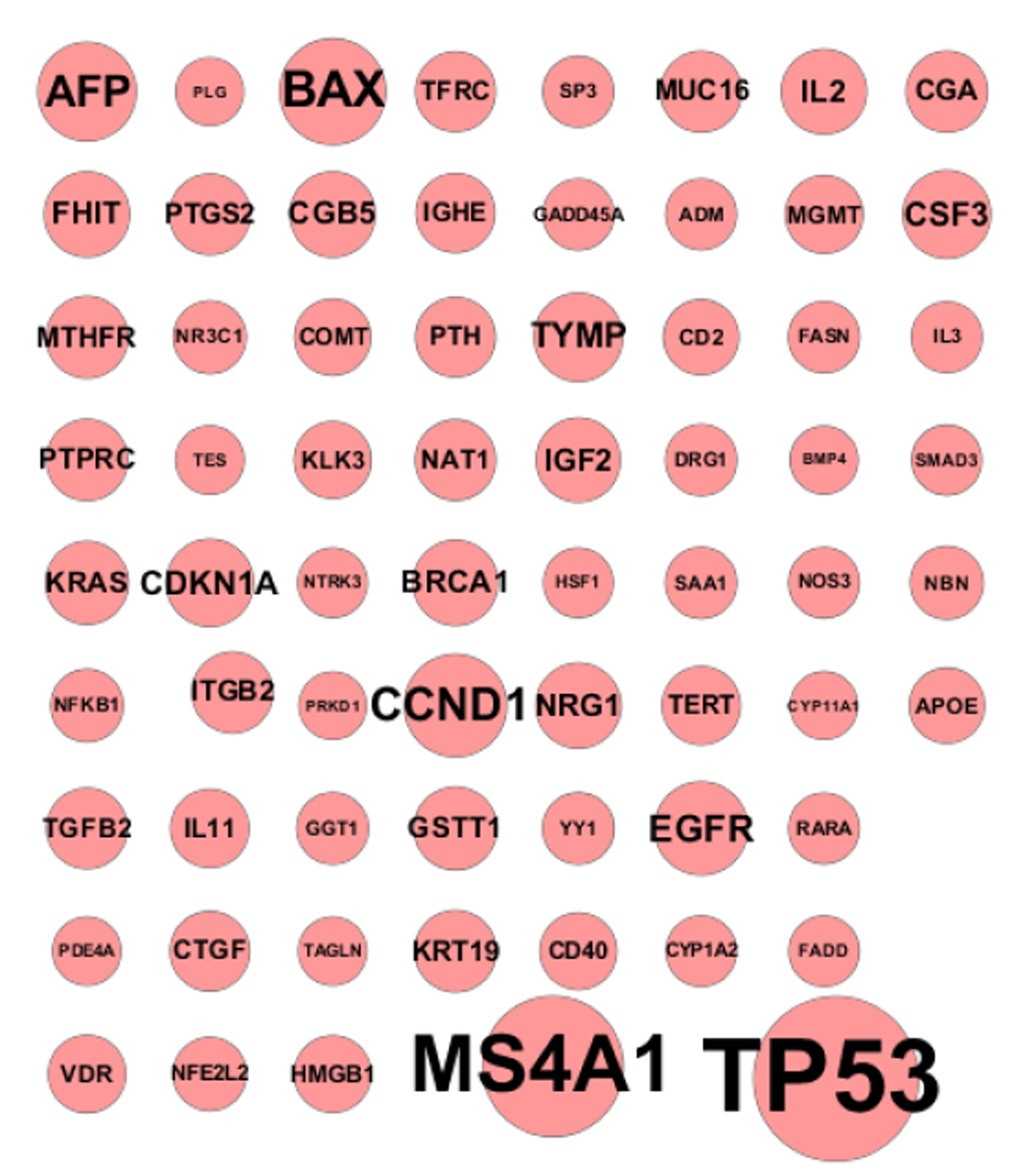

Supplement: Figure S14 — A cancer-association map of DTGs in prostate cancer. (TIF) [file pone.0040960.s014.tif]

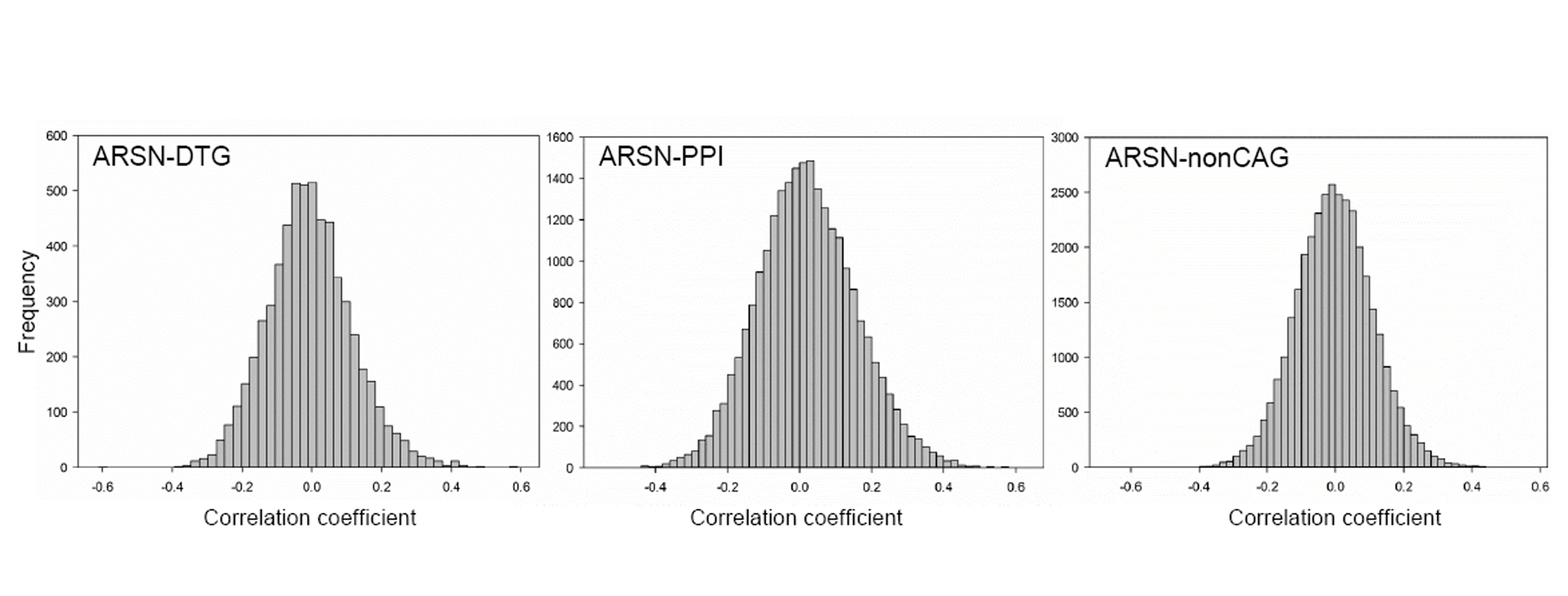

Supplement: Figure S15 — Histogram showing the frequency of the correlation coefficient. The histogram was computed from the three interaction set. Bars represent number of correlation coefficients within the range indicated on the x-axis (P<0.001, one-way ANOVA). (TIF) [file pone.0040960.s015.tif]

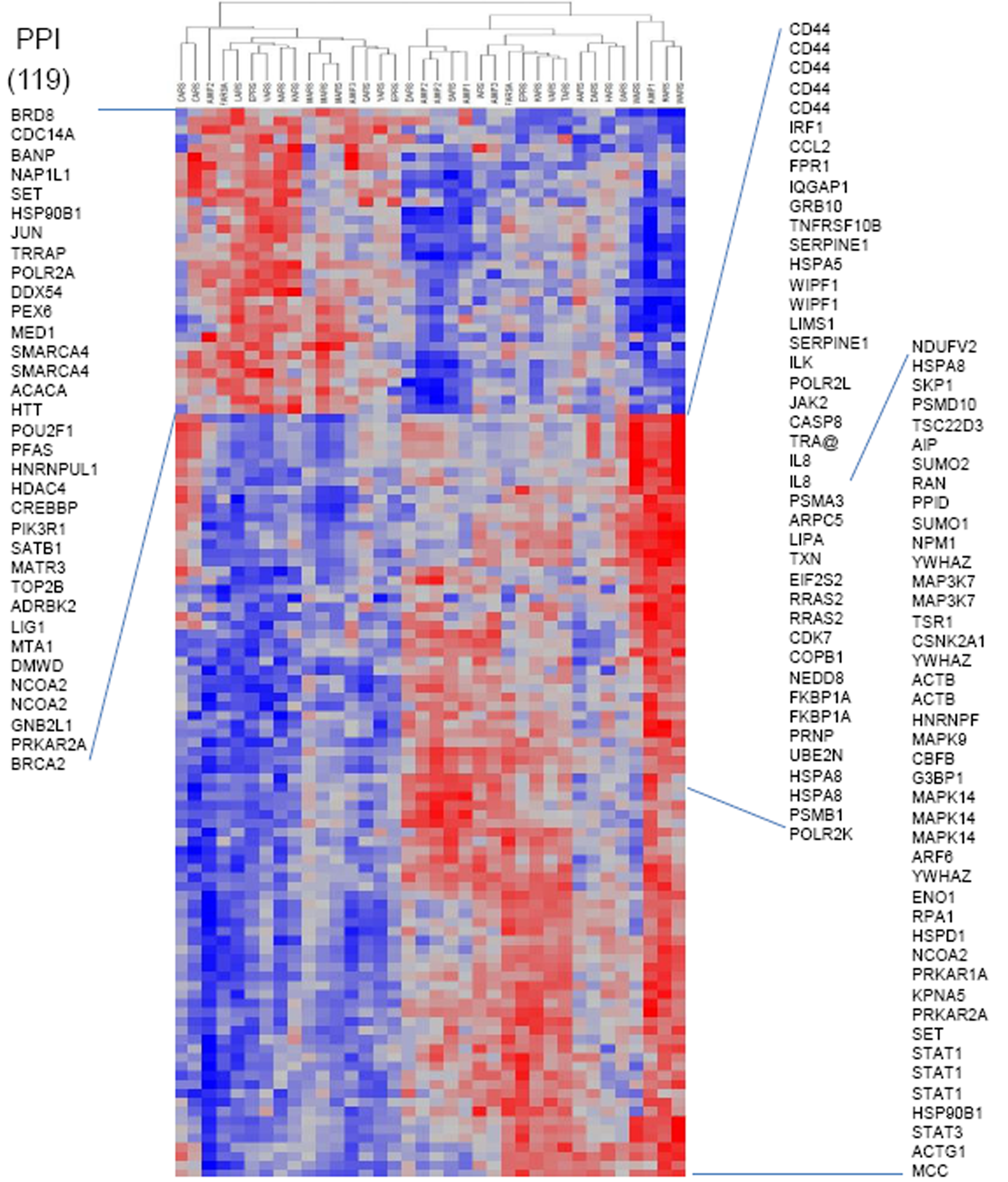

Supplement: Figure S16 — Correlation patterns of 23 ARSs and AIMPs to PPI. Hierarchical clustering analysis showed that ARSN were shared by two groups with 119 PPIs (FDR <0.014). 119 PPIs were generated on a supervised hierarchical clustering analysis. (TIF) [file pone.0040960.s016.tif]

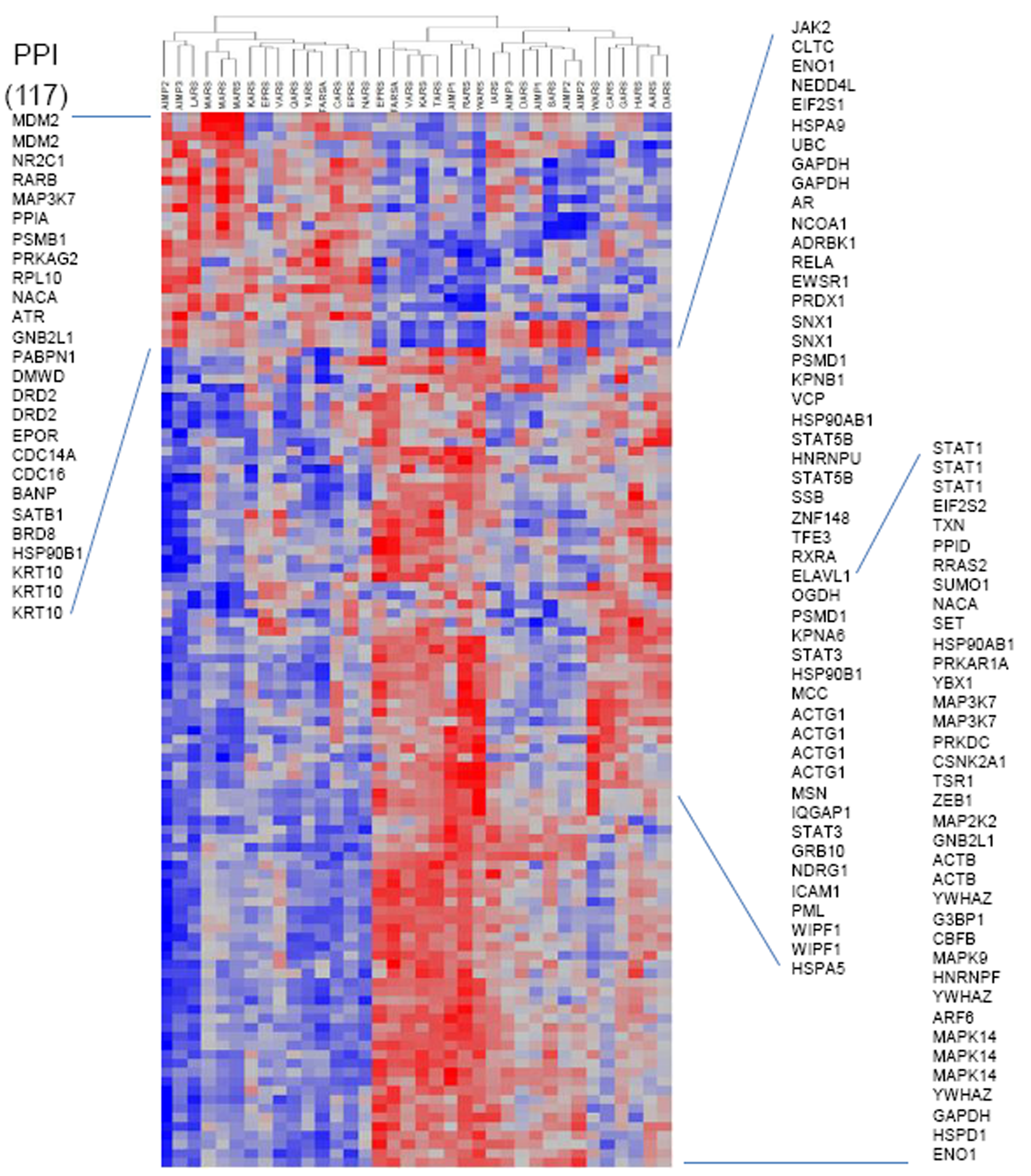

Supplement: Figure S17 — Correlation patterns of 23 ARSs and AIMPs to PPI. Hierarchical clustering of ARSN based on the 117 DTGs based on nonlinear association between two gene expression sets. 117 PPIs were correlated with two subgroups of ARSN. (TIF) [file pone.0040960.s017.tif]

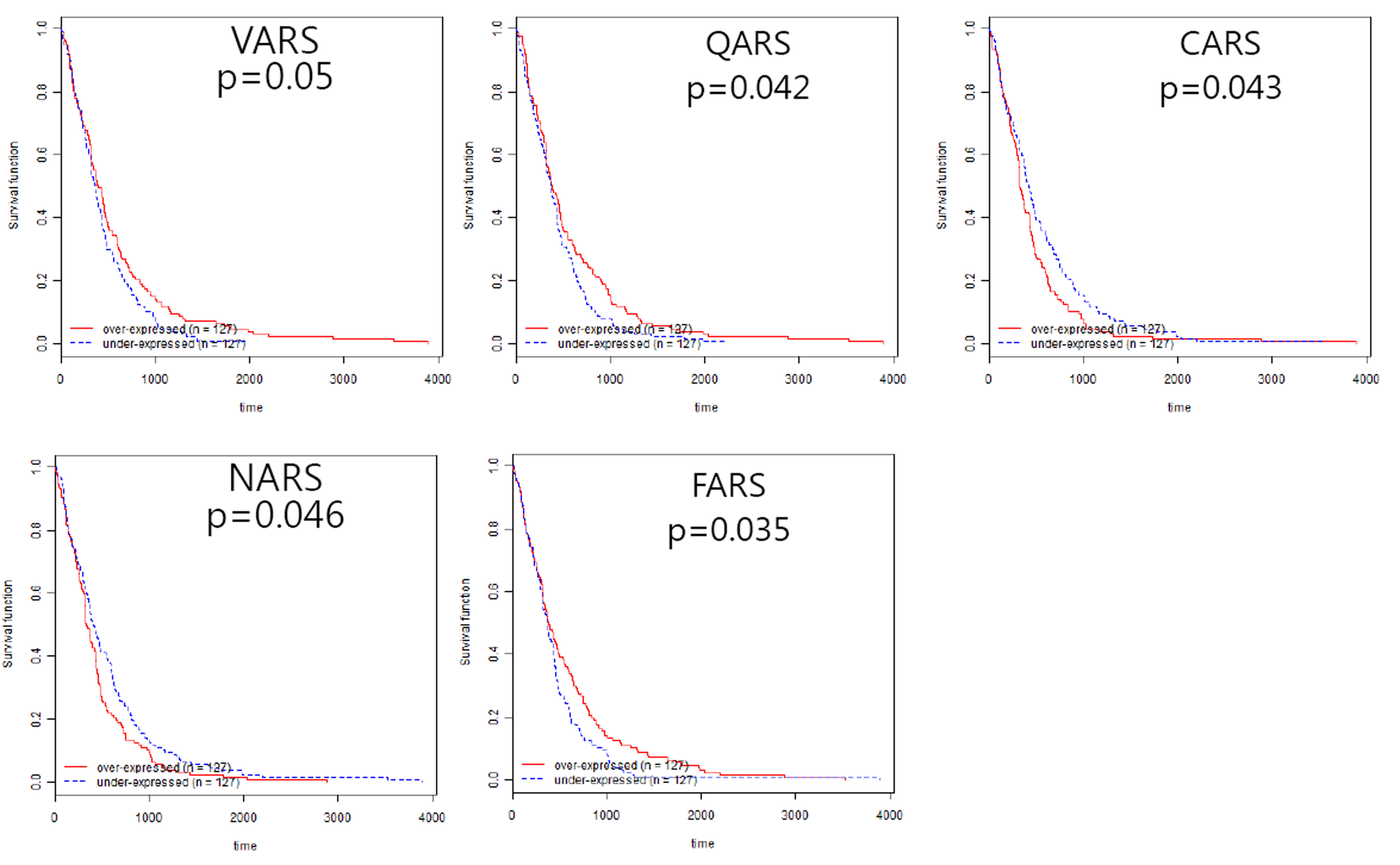

Supplement: Figure S18 — Effect of ARSN gene expression on survival in 254 GBM patients. Kaplan-Meier plot of overall survival in 254 GBM patients grouped on the basis of expression of each probeset. The difference between two groups was significant when the P value was less than 0.05. (TIF) [file pone.0040960.s018.tif]

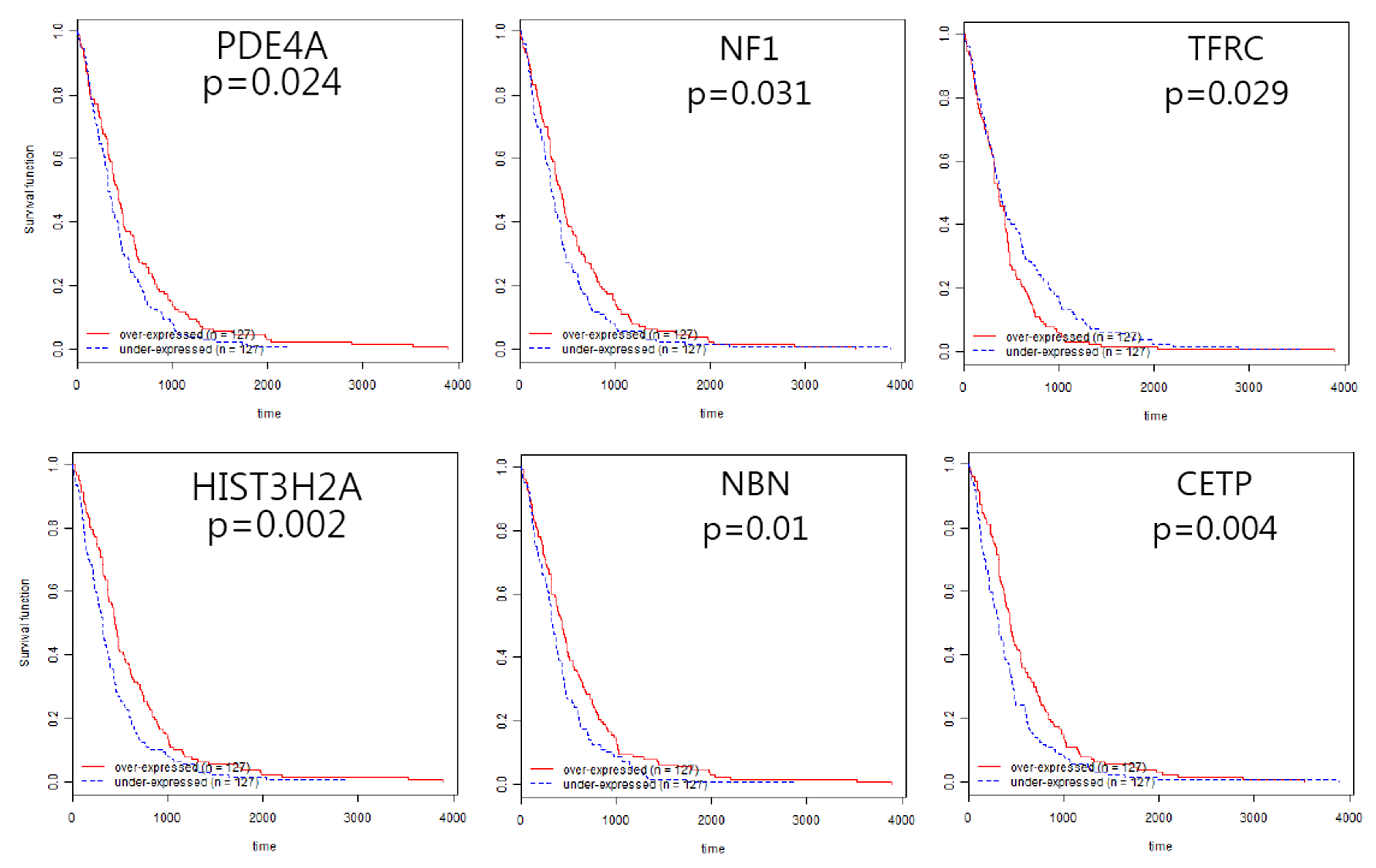

Supplement: Figure S19 — Effect of DTG gene expression on survival in 254 GBM patients. (TIF) [file pone.0040960.s019.tif]

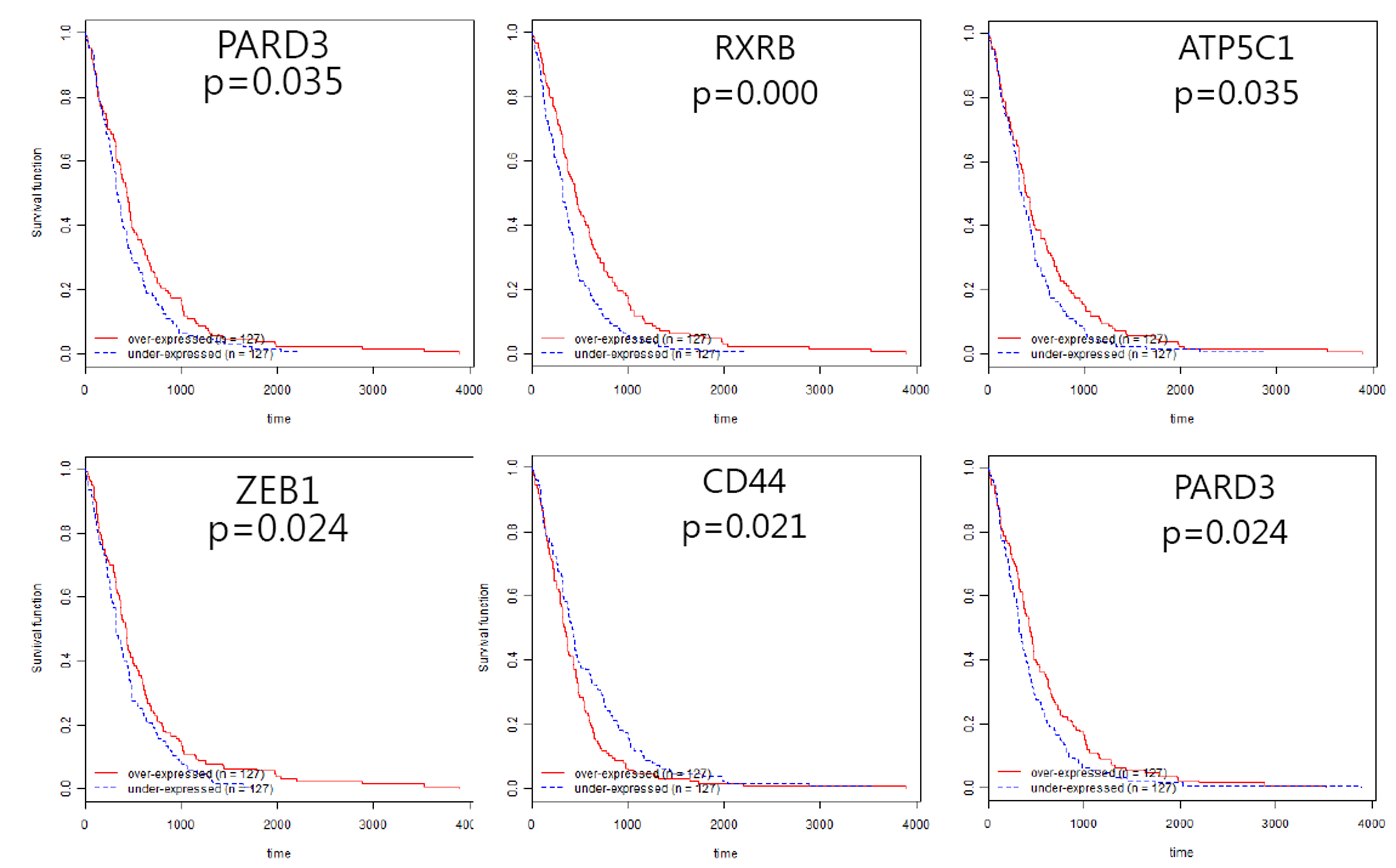

Supplement: Figure S20 — Effect of PPI gene expression on survival in 254 GBM patients. (TIF) [file pone.0040960.s020.tif]

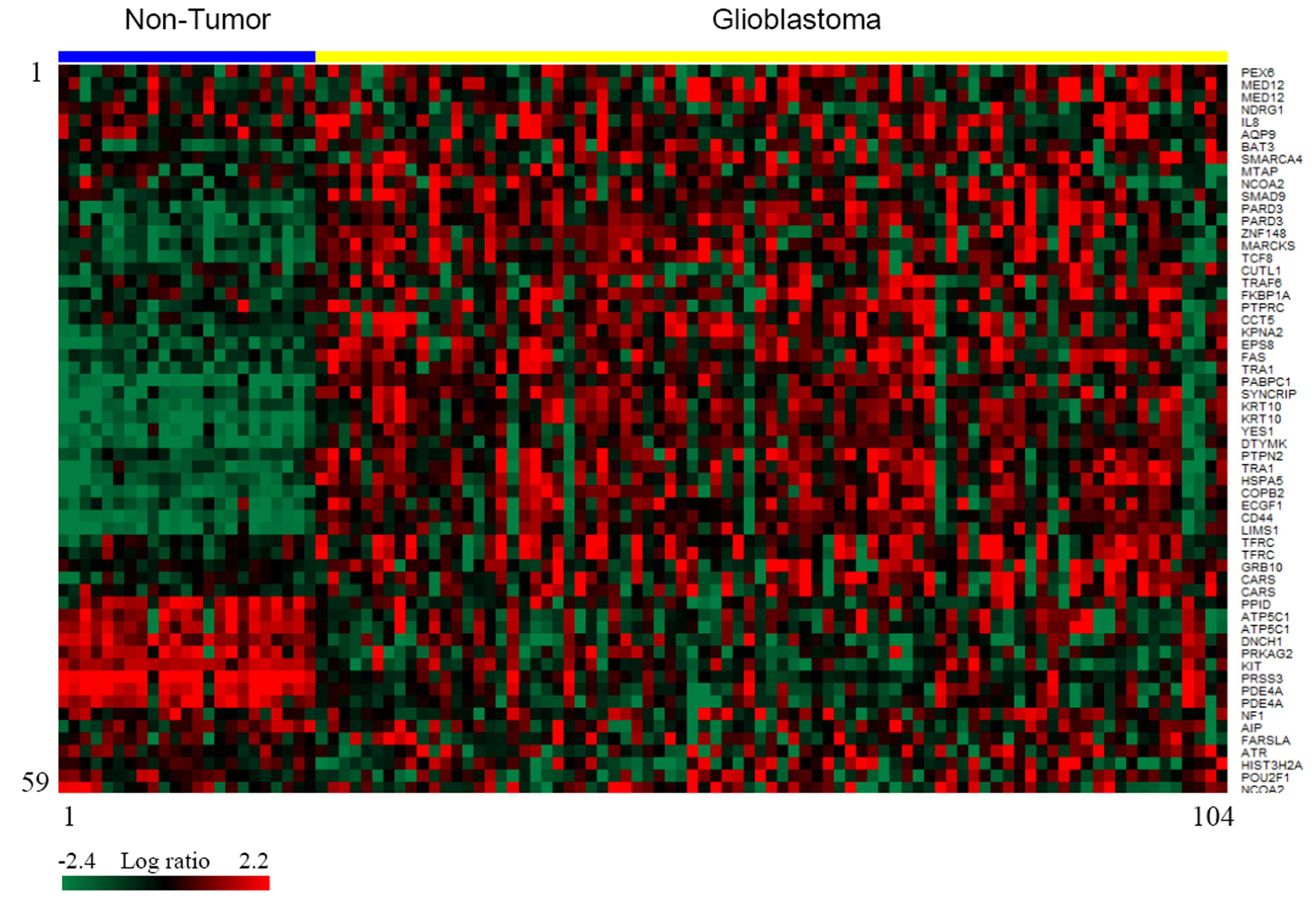

Supplement: Figure S21 — Hierarchical clustering of the GSE4290 dataset. Hierarchical clustering of the GSE4290 dataset of 81 GBM samples from patients with GBM and 23 non-tumor tissues based on the 61 probe sets. Nine probes were significantly overexpressed in the non-tumor samples, with 2 probes not showing in this analysis. The data are presented in matrix format in which rows represent individual genes and columns represent each tissue. Each cell in the matrix represents the expression level of a gene feature in an individual tissue. Red and green in cells reflect high and low expression levels, respectively. (TIF) [file pone.0040960.s021.tif]

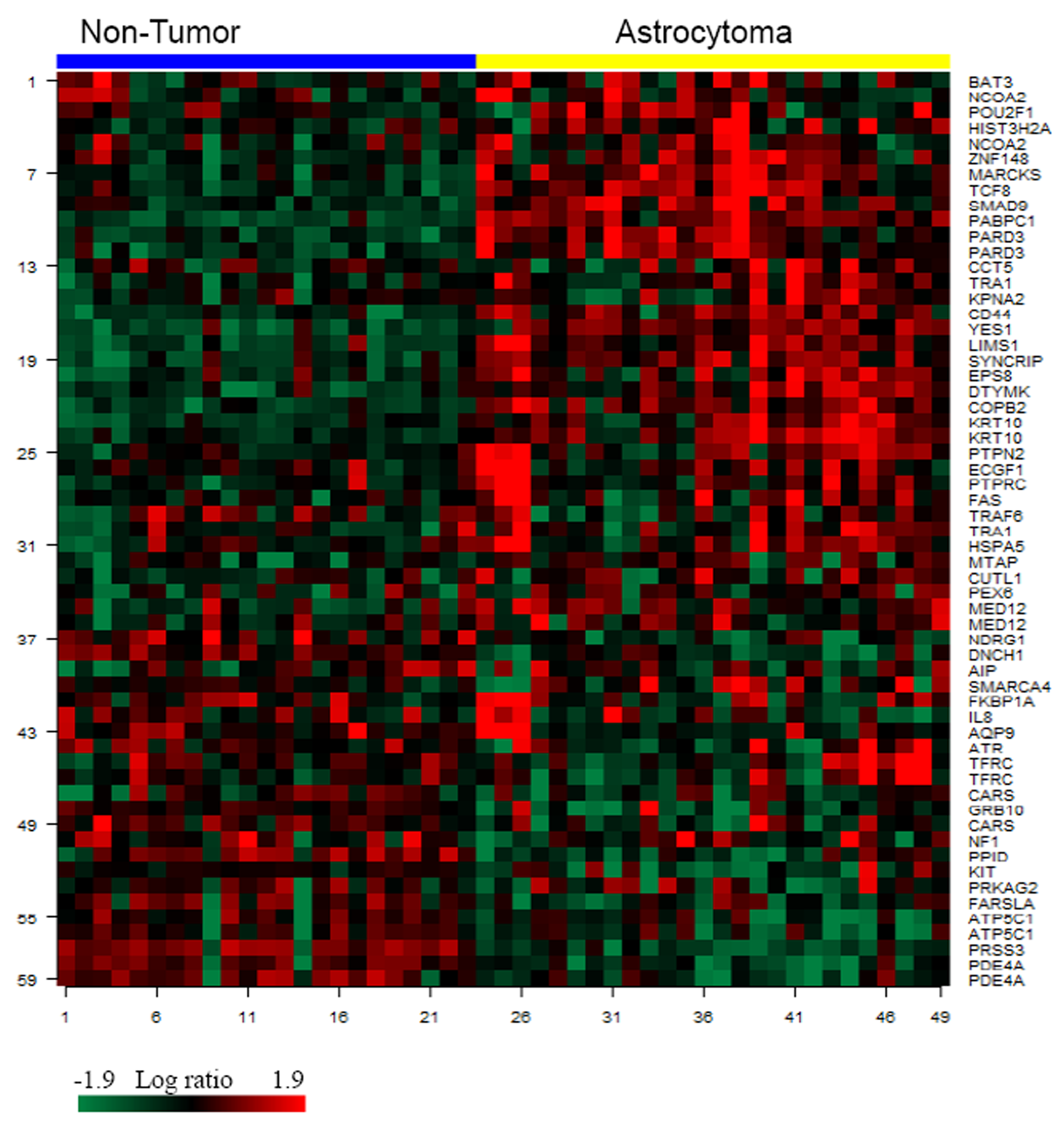

Supplement: Figure S22 — Hierarchical clustering of the GSE4290 dataset of astrocytomas (II and III). (TIF) [file pone.0040960.s022.tif]

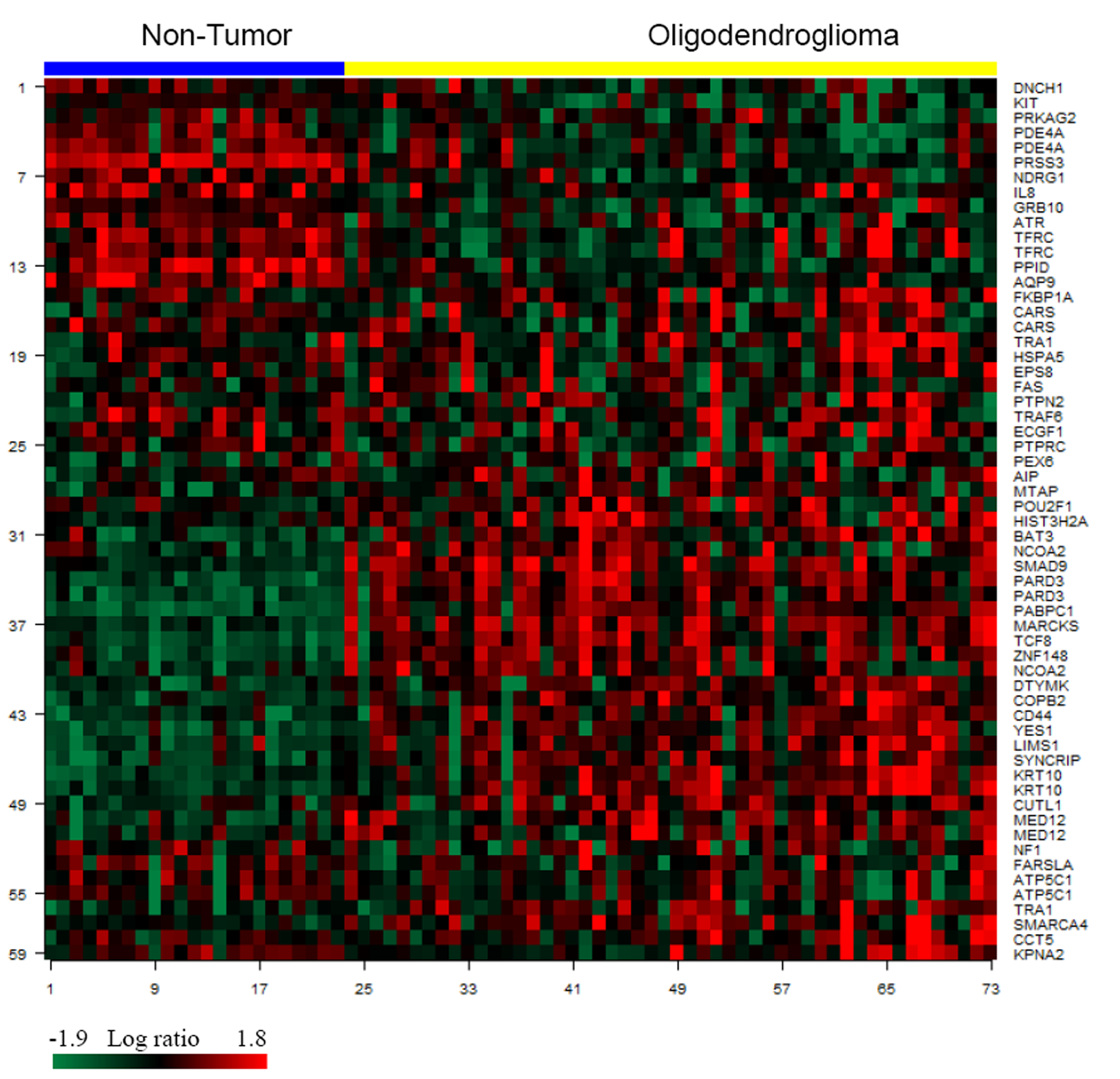

Supplement: Figure S23 — Hierarchical clustering of the GSE4290 data set of oligodendrogliomas (II and III). (TIF) [file pone.0040960.s023.tif]
